# The estrous cycle surpasses sex differences in regulating the transcriptome in the rat medial prefrontal cortex and reveals an underlying role of early growth response 1

**DOI:** 10.1186/s13059-015-0815-x

**Published:** 2015-12-02

**Authors:** Florian Duclot, Mohamed Kabbaj

**Affiliations:** Department of Biomedical Sciences, College of Medicine, Florida State University, 1115 W Call Street, Tallahassee, FL 32306 USA; Program in Neuroscience, College of Medicine, Florida State University, 1115 W Call Street, Tallahassee, FL 32306 USA

**Keywords:** Early growth response 1 (Egr1), Estrous cycle, Medial prefrontal cortex (mPFC), Ovarian hormones, Sex differences, Synapse, Transcriptome

## Abstract

**Background:**

Males and females differ in cognitive functions and emotional processing, which in part have been associated with baseline sex differences in gene expression in the medial prefrontal cortex. Nevertheless, a growing body of evidence suggests that sex differences in medial prefrontal cortex-dependent cognitive functions are attenuated by hormonal fluctuations within the menstrual cycle. Despite known genomic effects of ovarian hormones, the interaction of the estrous cycle with sex differences in gene expression in the medial prefrontal cortex remains unclear and warrants further investigations.

**Results:**

We undertake a large-scale characterization of sex differences and their interaction with the estrous cycle in the adult medial prefrontal cortex transcriptome and report that females with high and low ovarian hormone levels exhibited a partly opposed sexually biased transcriptome. The extent of regulation within females vastly exceeds sex differences, and supports a multi-level reorganization of synaptic function across the estrous cycle. Genome-wide analysis of the transcription factor early growth response 1 binding highlights its role in controlling the synapse-related genes varying within females.

**Conclusions:**

We uncover a critical influence of the estrous cycle on the adult rat medial prefrontal cortex transcriptome resulting in partly opposite sex differences in proestrus when compared to diestrus females, and we discovered a direct role for Early Growth Response 1 in this opposite regulation. In addition to illustrating the importance of accounting for the estrous cycle in females, our data set the ground for a better understanding of the female specificities in cognition and emotional processing.

**Electronic supplementary material:**

The online version of this article (doi:10.1186/s13059-015-0815-x) contains supplementary material, which is available to authorized users.

## Background

In both humans and rodents, males and females greatly differ on a variety of levels, from brain morphology to function, leading to discrete differences in high-order processes such as cognitive functions and emotional responses [1–5]. Although a causal relationship remains to be determined, the search for neurobiological correlates revealed clear sex differences in gene expression profiles in several brain areas such as the medial prefrontal cortex (mPFC) [6–8], which plays a central role in cognitive functions.

The importance of sex differences in the mPFC and mPFC-dependent processes can be observed both at the morphological and neurophysiological levels. Indeed, sexual dimorphisms in brain volumes are most pronounced in the mPFC, with men and women having larger volumes in the frontomedial cortex and dorsolateral cortex, respectively [5]. Moreover, neuronal activity in the mPFC also exhibits gender differences, as activation of the anterior cingulate and prefrontal cortices to negative stimuli is greater in women than men [6]. Furthermore, recent rodents’ studies revealed that mPFC neurons encode the anxiogenic nature of an environment through synchrony with the ventral hippocampus, whereas they encode exploratory behavior through synchrony with the basolateral amygdala [7]. The mPFC therefore controls a variety of high-order processes such as emotional processing or cognitive functions, and is at the center of clear sex differences at the morphological and neurophysiological levels, which suggests that sex differences in gene expression in the mPFC likely underlie sex differences in mPFC-dependent processes. In line with this hypothesis, we previously reported that the expression levels of the immediate early gene early growth response 1 (*Egr1*) in the rat mPFC control sex differences in social anxiety behaviors [8].

Notably, the sexual dimorphism in mPFC-dependent processes is attenuated by hormonal fluctuations throughout a woman’s reproductive life or within the menstrual cycle [9, 10]. Indeed, a growing body of evidence suggests that fluctuations in circulating levels of estrogens in postmenopausal women or healthy cycling women can affect PFC-dependent working memory and executive functions [4, 11–14]. Surprisingly, despite the vast genomic effects of ovarian hormones in the rodent mPFC [15], the effect of the estrous cycle on the mPFC transcriptome and subsequent interaction with cognitive functions and emotional processing remain to be investigated.

It therefore appears critical to first characterize sex differences in gene expression in the mPFC and their alteration by the estrous cycle. To this aim, we first undertook a large-scale transcriptomic approach by RNA-sequencing (RNA-seq) in rats to compare the gene expression profiles in the mPFC of males, proestrus females, and diestrus females, thereby accounting for fluctuations in sex hormone levels (high in proestrus, low in diestrus). Then, because sex differences in social anxiety in rats are controlled by Egr1 [8], we sought to discover the specific genes under the direct transcriptional control of Egr1 by chromatin immunoprecipitation followed by sequencing (ChIP-seq). This approach thus allowed the identification of a distinct and partly opposite sexually biased transcriptome in the mPFC of proestrus and diestrus females and its underlying control by Egr1. Furthermore, the extent of differences in gene expression and alternative splicing events were far larger between females than between sexes, and revealed specific functional pathways affected by the estrous cycle.

## Results

### The estrous cycle influences sexually biased gene expression

In adult organisms, while substantial evidence points towards a large transcriptomic sex bias in reproductive tissues, differences in the brain are more specific [16–18]. Moreover, despite widespread genomic regulation by the female sex hormones estrogen and progesterone [15], the transcriptomic regulation across the estrous cycle is still unknown, which led us to first examine the overall gene expression profile of males, proestrus females, and diestrus females.

**Fig. 1:**
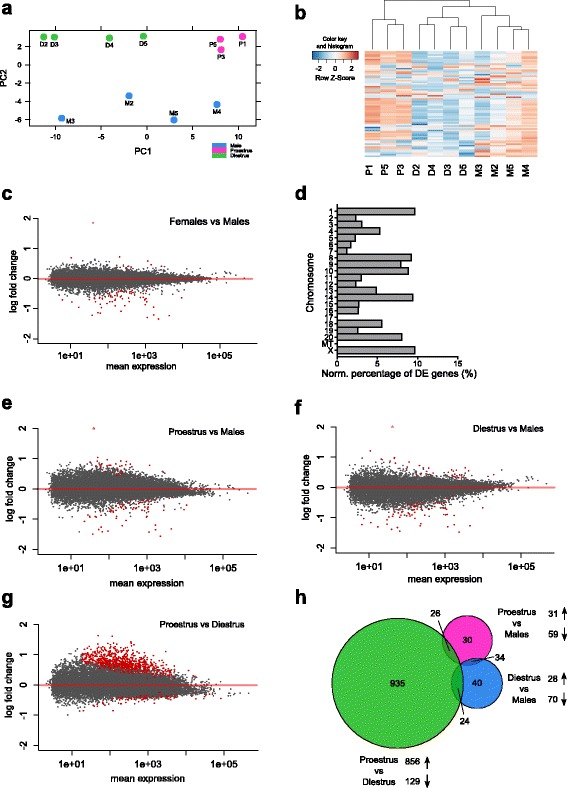
A sexually biased transcriptome in the rat medial prefrontal cortex (mPFC) and effect of the estrous cycle. **a** A principal component analysis separates males and females along the second axis while proestrus and diestrus females are clustered separately along the first axis. **b** The hierarchical clustering of the 70 genes showing the most variance (rlog-transformed) revealed more similarity of diestrus females to males over proestrus females. **c**, **e**-**g** Representation of the log2 fold-change over the averaged normalized read counts, with significantly different genes at the false discovery rate (FDR) 5 % threshold highlighted in *red*. **d** The sexually dimorphic genes in the rat mPFC do not show a sex-chromosome bias, and are distributed among all chromosomes. To account for differences in number of genes per chromosome, data were normalized to the total number of genes detected in our study on each chromosome. **h** The Venn diagram representing the number of differentially expressed genes (FDR 5 %) in all pairwise comparisons depicts a relatively small overlap between genes affected by the estrous cycle within females, and those sexually biased in either stage of the cycle. The area of each circle is proportional to the number of genes it contains. In (**a**–**c**, **e**-**g**), values from the R package DESeq2 were used

While males were distinct from females, a clear separation was also noted between proestrus and diestrus females following principal component analysis (Fig. 1a). Moreover, based on the genes with the most variance, diestrus females were clustered more closely to males than to proestrus females (Fig. 1b). To better assess the impact of the estrous cycle, we first sought to identify and compare the differentially expressed genes (DEG) between males and females with or without accounting for the estrous cycle. Without discriminating for the estrous cycle, 67 of the 15,607 genes detected in our study survived the 5 % false discovery rate (FDR) threshold, of which 91 % were down-regulated in females (Fig. 1c; Additional file 1: Figure S1a, e), and were distributed throughout the genome (Fig. 1d), suggesting that sex differences in the adult rat transcriptome cannot be solely explained by sex chromosomes.

When discriminating between proestrus and diestrus, we observed a comparable extent of sex differences, as both female groups exhibited a similar number of DEG versus males when compared separately (proestrus versus males, diestrus versus males) than when grouped together (females versus males). When compared to males, proestrus and diestrus females exhibited 90 and 98 DEG with 66 % and 71 %, respectively, down-regulated (Fig. 1e, f; Additional file 1: Figure S1b, c, e). Within females, however, differences were larger, with 985 DEG between proestrus and diestrus and a majority (87 %) being up-regulated in proestrus (Fig. 1g, Additional file 1: Figure S1d, e; Additional file 2: Table S1). Together with the relatively small overlap observed between each pairwise comparison (Fig. 1h), this denotes a profound reorganization of the transcriptome throughout the estrous cycle that exceeds sex differences and suggests the involvement of distinct pathways in each biological group. Nevertheless, no substantial differences were observed in the amplitude of fold-change between each pairwise comparison (Additional file 1: Figure S1f), revealing that the extent but not the intensity of gene regulation is larger within females than between sexes.

### Alterations in cellular communication in the female rat mPFC

**Fig. 2:**
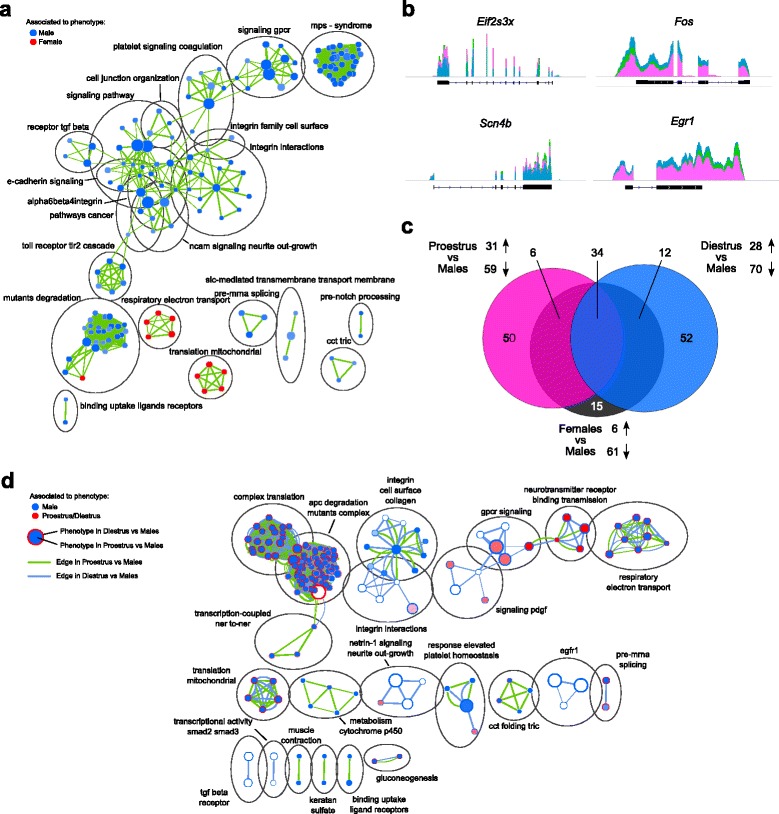
Sexually biased genes in the rat medial prefrontal cortex (mPFC) are associated with cellular communication and translation. **a** Enrichment map depicting the clusters of differentially modulated pathways between females and males identified by the gene-set enrichment analysis. The area of each node, representing a gene set (functional pathway), corresponds to the number of genes of the gene set it contains, and the edge thickness is proportional to the number of genes overlapping between the two connected nodes. Pathways related to the interaction with the extracellular matrix and its downstream signaling were widely associated with males, while only pathways related to translation and oxidative phosphorylation were associated with the female phenotype. **b** Illustration of the averaged read coverage for the male (*blue*), proestrus (*pink*), and diestrus (*green*) groups for two genes up-regulated (*left*) or down-regulated (*right*) in females when compared to males. **c** A substantial proportion of the genes differentially expressed in proestrus or diestrus when compared to males are specific to each cycle stage. **d** Enrichment map depicting the cluster of pathways identified by gene-set enrichment analysis as differentially regulated in the proestrus versus males (*inner circle* of each node), and diestrus versus males comparisons (*outer ring* of each node). *Green* and *blue* edges correspond to the proestrus versus males, and diestrus versus males datasets, respectively

To analyze the functional implications of the sexually biased transcriptome in the rat mPFC, we first conducted a gene-set enrichment analysis (GSEA) comparing males to females without accounting for the estrous cycle. In line with the preponderance of down-regulation in females (Fig. 1c; Additional file 1: Figure S1a, e), the vast majority of phenotypes were preferentially associated with males (Fig. 2a). While only processes related to mitochondrial oxidative phosphorylation and translation were enriched in females, males showed a vast enrichment of interaction with the extracellular matrix (ECM) and its downstream signaling (integrin family, cadherin signaling, cell–cell junction, Ncam signaling). Furthermore, all genes tested for verification by semi-quantitative real-time PCR confirmed the up-regulation (*Rgs9*, *Gucy1b2*, *Eif2s3x*, *Scn4b*) or down-regulation (*Fos*, *FosB*, *BDNF*, *Igf2*, *Egr1*) observed in females over the males without estrous cycle interaction (Fig. 2b; Additional file 3: Figure S2). However, although an overall reduction of *Egr1* in females was confirmed (Additional file 4: Table S2), its levels were reduced in proestrus only (Additional file 3: Figure S2b). Such estrous cycle-specific effect was particularly noticeable when comparing sexually biased DEG in either proestrus or diestrus. Indeed, only a 46 % and 47 % overlap was observed, respectively (Fig. 2c), revealing that the majority of sexually biased genes in the mPFC were distinct in proestrus from diestrus.

Accordingly, the GSEA of the sexually biased genes with discrimination between proestrus and diestrus revealed an opposite regulation of processes related to translation, degradation, and oxidative phosphorylation between the two female groups when compared to males (Fig. 2d). Interestingly, the only processes up-regulated in proestrus and down-regulated in diestrus, when compared to males, were related to neurotransmission and its downstream signaling, suggesting proestrus-specific sex differences in synaptic transmission. However, both estrous cycle stages exhibited a down-regulation of genes associated with ECM organization (integrin-related terms), although they were more pronounced in diestrus. Furthermore, functional clustering of the 40 DEG only in diestrus versus males revealed a weak enrichment for ion transport-related processes (Table 1), whereas the 30 DEG only in proestrus versus males displayed an enrichment of transcriptional processes mainly carried out by the transcription factor *Egr1* (Table 2; Additional file 5: Figure S3).

**Table 1:** Annotation clusters enriched in sexually biased genes specific to diestrus

Cluster enrichment score	Term	Count	*p*-value	Fold enrichment
Cluster #1: 2.06	Metal ion binding	13	3.04E−03	2.27
	Cation binding	13	3.43E−03	2.24
	Ion binding	13	4.00E−03	2.20
	Transition metal ion binding	7	1.39E−01	1.86
Cluster #2: 1.51	Proteinaceous extracellular matrix	4	1.10E−02	8.16
	Extracellular region part	6	1.38E−02	3.89
	Extracellular matrix	4	1.58E−02	7.13
	Extracellular space	4	8.52E−02	3.65
	Extracellular region	6	1.29E−01	2.10
Cluster #3: 0.90	Sodium ion transport	3	2.27E−02	12.33
	Monovalent inorganic cation transport	3	1.28E−01	4.63
	Ion transport	4	1.71E−01	2.68
	Metal ion transport	3	2.14E−01	3.33
	Cation transport	3	2.86E−01	2.73

Only clusters with at least one term with *p*-value < 0.05 are represented

**Table 2:** Top 2 annotation clusters enriched in proestrus versus males

Cluster enrichment score	Term	Count	*p*-value	Fold enrichment
Cluster #1: 1.45	Negative regulation of transcription, DNA-dependent	5	5.41E−03	6.55
	Negative regulation of RNA metabolic process	5	5.71E−03	6.45
	Negative regulation of transcription	5	1.20E−02	5.20
	Negative regulation of transcription from RNA polymerase II promoter	4	1.51E−02	7.22
	Negative regulation of gene expression	5	1.61E−02	4.77
	Negative regulation of nucleobase, nucleoside, nucleotide, and nucleic acid metabolic process	5	1.73E−02	4.68
	Negative regulation of nitrogen compound metabolic process	5	1.82E−02	4.60
	Negative regulation of macromolecule biosynthetic process	5	2.09E−02	4.42
	Negative regulation of cellular biosynthetic process	5	2.23E−02	4.33
	Negative regulation of biosynthetic process	5	2.41E−02	4.23
	Negative regulation of macromolecule metabolic process	5	5.54E−02	3.25
	Regulation of transcription from RNA polymerase II promoter	4	1.48E−01	2.85
	Transcription	4	2.03E−01	2.45
	Regulation of transcription, DNA-dependent	5	2.23E−01	1.96
	Regulation of RNA metabolic process	5	2.40E−01	1.90
	Regulation of transcription	6	2.41E−01	1.70
Cluster #2: 0.57	Regulation of growth	3	1.57E−01	4.04
	Negative regulation of apoptosis	3	1.78E−01	3.74
	Negative regulation of programmed cell death	3	1.81E−01	3.70
	Negative regulation of cell death	3	1.82E−01	3.68
	Regulation of apoptosis	3	4.74E−01	1.82
	Regulation of programmed cell death	3	4.81E−01	1.80
	Regulation of cell death	3	4.83E−01	1.79

Altogether, our observations highlight alterations in ECM organization and downstream signaling pathways between males and females, suggesting differences in cellular communication in the mPFC. Nevertheless, proestrus females showed a specific alteration of neurotransmission-related genes, as well as transcriptional-related processes mainly carried by *Egr1*, suggesting a particular role for this immediate early gene in the sex differences observed in proestrus.

#### Widespread reorganization of the rat mPFC transcriptome between proestrus and diestrus

**Fig. 3:**
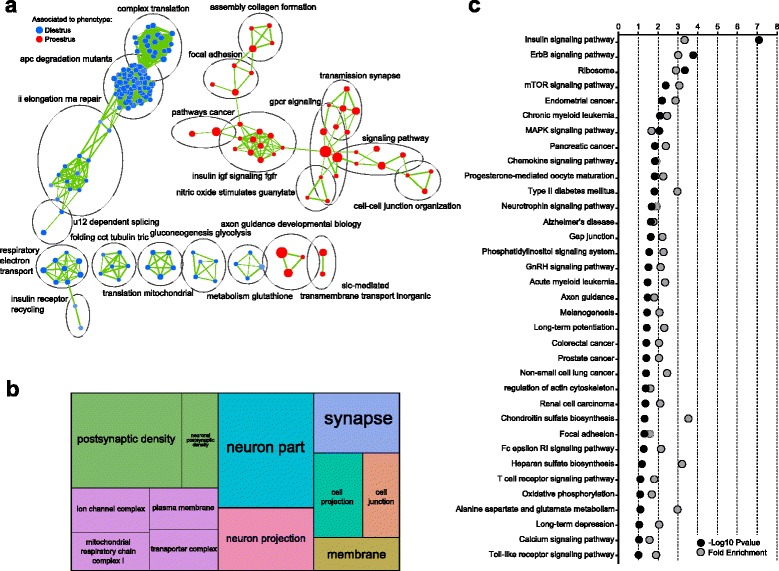
Widespread functional reorganization of the rat medial prefrontal cortex transcriptome throughout the estrous cycle. **a** Enrichment map depicting the clusters of differentially modulated pathways between proestrus and diestrus females identified by the gene-set enrichment analysis. The area of each node, representing a gene set (functional pathway), corresponds to the number of genes of the gene set it contains, and the edge thickness is proportional to the number of genes overlapping between the two connected nodes. Pathways related to translation, degradation, oxidative phosphorylation, and transcription are associated with diestrus females, whereas extracellular matrix interactions, as well as insulin and synaptic signals transduction are associated with proestrus females. **b** treemap representation of Cellular Compartment terms from the Gene Ontology database, showing a marked enrichment of neuronal and synaptic genes. The size of each rectangle is proportional to the -log10 of the *p*-value (the bigger the rectangle, the more significant the enrichment). **c** The enrichment analysis of pathways from the Kyoto Encyclopedia of Genes and Genomes reveals a widespread alteration of signal transduction pathways, including neuronal and synaptic, as well as translation processes

In line with the distinct sex bias between proestrus and diestrus, widespread differences in gene expression (985 DEG) were observed between these two stages of the estrous cycle. Following GSEA, we observed an up-regulation of processes related to translation, degradation, and oxidative phosphorylation in diestrus (Fig. 3a), confirming our examination of sex-biased DEG (Fig. 2d), alongside a robust representation of DNA transcription consistent with the enrichment of transcription-related terms in proestrus-specific sex-biased DEG (Table 2). Supporting our previous observation (Fig. 2d), neurotransmission-related terms were widely enriched in proestrus when compared to diestrus. Indeed, cell–cell junction, neuronal organization, synaptic transmission and general signaling pathways were found enriched and associated with proestrus (Fig. 3a), and strengthened by a large enrichment of Gene Ontology (GO) terms related to the synaptic compartment of the cell (Fig. 3b; Additional file 6: Table S3). Similarly, the enrichment analysis of terms from the Kyoto Encyclopedia of Genes and Genomes (KEGG) revealed alterations in insulin signaling, translation, synaptic transmission, and general signaling pathways (Fig. 3c), supporting global differences in signal transduction. Interestingly, these genes were found to be significantly associated with pathologies such as depression, behavioral disease, and bipolar disorder, unlike DEG in other conditions (Additional file 7: Figure S4), denoting the functional importance and relevance of the mPFC transcriptome reprogramming throughout the estrous cycle.

Furthermore, driven by our observations of sexually biased expression in mRNA splicing genes (Figs. 2a, d and 3a) and the fact that alternative splicing events are extensively observed in the mammalian brain, and are sexually dimorphic [18, 19], we conducted an analysis of differential exon usage accounting for differences in gene expression, which thus allowed the assessment of alternative transcription start site and polyadenylation site usage in addition to alternative splicing [20]. Similar to our gene expression results, extensive regulations were present within females, but moderate between sexes, denoting a large impact of the estrous cycle on alternative splicing events (Additional file 8: Table S4). Interestingly, particular changes in synaptic assembly and vesicular transport between proestrus and diestrus were detailed (regulation of actin cytoskeleton, dendritic spine, neuron spine, vesicle; Additional file 9: Figure S5).

Our observations highlight alterations in ECM organization and downstream signaling between males and females, supporting differences in cellular communication in the mPFC. Nevertheless, proestrus females showed a specific enrichment of neurotransmission, as well as transcription-related processes carried mainly by *Egr1*, suggesting a role for this immediate early gene in the sex differences observed in proestrus.

#### Variations in synaptic functions are preponderant among sex and estrous cycle regulations

We observed substantial variations in the rat mPFC transcriptome, both by sex and by the stage of the estrous cycle. However, despite a wider range of variations between females than between sexes, the respective impact of each factor (sex and estrous cycle), and more importantly their interaction, remained to be investigated. To this end, we clustered all genes based on their pattern of regulation between all three biological conditions, allowing for the identification of genes being affected by sex (proestrus and diestrus similar to each other, but different from males), estrous cycle (either proestrus or diestrus different from males), or both (both proestrus and diestrus different from males and from each other).

**Fig. 4:**
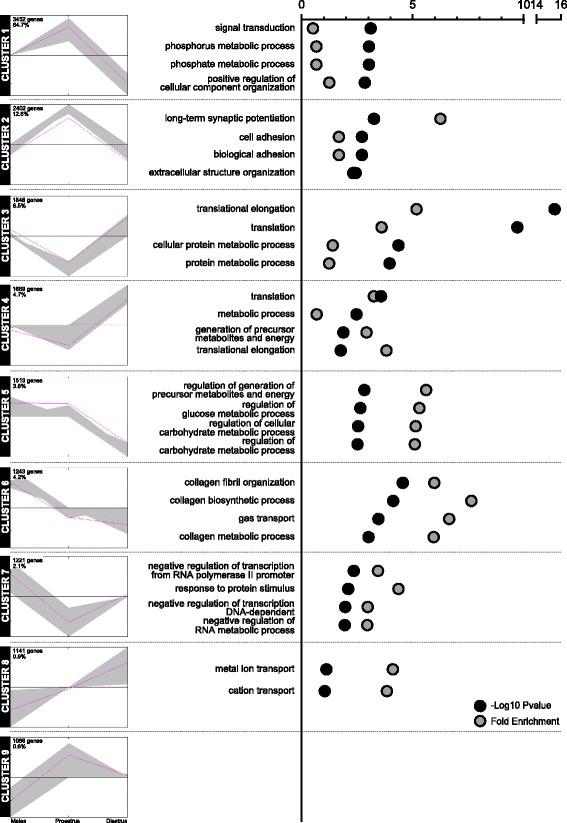
Estrous cycle-dependent transcriptomic regulation exceeds sex differences and primarily targets synaptic function. A gene clustering analysis identified nine distinct profiles of gene regulation between males (*left side*), proestrus (center), and diestrus females (*right side*). At the top left corner of each plot is detailed the total number of genes as well as the percentage of all differentially expressed genes represented in the cluster. In each cluster, the enrichment in Biological Processes from the Gene Ontology (GO) database was analyzed for the differentially expressed genes represented in the cluster. The first four hits (if four or more hits were found) ranked by *p*-value are depicted on the right of each plot and highlight the enrichment of synapse-related genes in proestrus females from clusters 1 and 2, translation processes in diestrus females from clusters 3 and 4, and interaction with the extracellular matrix from cluster 6. The full list of enriched GO terms can be found in Additional file 10: Table S5

Out of the nine optimal clusters identified (Fig. 4), four showed regulation by the estrous cycle alone (representing 48 % of all genes detected), three displayed regulation by both sex and estrous cycle (37 % of all genes), whereas only two showed an effect of sex alone (15 % of all genes). Notably, 68 % and 28 % of all DEG showed regulation by both sex and estrous cycle or by the estrous cycle alone, respectively, while only 5 % were affected by sex alone, thereby demonstrating further the profound impact of the estrous cycle on the rat mPFC transcriptome. In particular, almost two thirds of all DEG (64.7 %) were up-regulated in proestrus when compared to males, and down-regulated in diestrus to reach lower levels than males (Fig. 4, cluster 1). The second biggest proportion of DEG showed a similar up-regulation in proestrus, but returned to the levels of males in diestrus (Fig. 4, cluster 2). Interestingly, the DEG present in these two main clusters were primarily associated with synaptic organization, function, and signal transduction, thereby revealing the preponderant nature of the synaptic regulation and its modulation by both sex and estrous cycle (Additional file 10: Table S5). The remaining clusters highlighted the less pronounced alterations in translation, up-regulated in diestrus when compared to either proestrus (Fig. 4, cluster 3) or males (Fig. 4, cluster 4), as well as the down-regulation of genes associated with ECM organization in females regardless of the estrous cycle (Fig. 4, cluster 6). Finally, we also confirmed the small but significant sex bias in the down-regulation of transcription-related genes, including *Egr1*, specific to proestrus (Fig. 4, cluster 7).

To investigate the contribution of the different cell types present in the rat mPFC to the profiles of gene expression regulated by sex and the estrous cycle, we analyzed the enrichment of different neuronal, glial, and other cell types in the DEG in each pairwise comparison. In line with the preponderance of variations in synaptic transmission among the estrous cycle regulations, both the “neuron” and “interneuron” cell types were enriched in the DEG between proestrus and diestrus females (Additional file 11: Figure S6a; Additional file 12: Figure S7a). Accordingly, the functional analysis of these “neuronal” genes revealed widespread associations with synaptic transmission, cell–cell communication, and intracellular signaling pathways (Additional file 11: Figure S6d, e; Additional file 12: Figure S7e), supporting the widespread alterations in synaptic transmission between proestrus and diestrus females. When comparing males to females, however, our analyses suggest a contribution of the “mural” cell type (pericytes and vascular smooth muscles), together with endothelial cells to a lesser extent, mainly carried by genes related to ECM interaction (Additional file 11: Figure S6a–c; Additional file 12: Figure S7a, c). It is important to note, however, that these sex differences appeared more pronounced in proestrus than in diestrus, as the enrichment in the mural or endothelial cell type failed to reach significance when analyzing DEG between diestrus and males. Interestingly, oligodendrocytes appeared to contribute to both sex and estrous cycle regulations in a maturation-dependent manner, with genes related to cell–cell adhesion and ECM interaction (Additional file 12: Figure S7a, b, d, f).

#### *Egr1 binds to synaptic plasticity genes in a sex-specific and estrous cycle-specific manner*

The pattern of sexually biased genes in the rat mPFC, distinct between proestrus and diestrus, included the down-regulation of transcription-related genes, mainly involving the transcription factor *Egr1* (Table 2; Fig. 4, cluster 7), and lower *Egr1* mRNA levels specific to proestrus (Fig. 2b; Additional file 3: Figure S2b). When analyzing transcription factors associated with our DEG in the literature, *Egr1* ranked among the top hits and was the top hit in the proestrus versus males comparison (Additional file 13: Table S6), where transcription-related terms carried by *Egr1* were enriched (Table 2). Within females, however, *Egr1* only ranked at the fifth position, likely owing to the high number of DEG. Interestingly, only *Egr1* and *Klf4* expression was sexually biased in the rat mPFC (Additional file 2: Table S1), and *Egr1* was the only candidate transcription factor with proestrus-specific gene regulation (Additional file 3, Figure S2b). Altogether, these observations suggest that *Egr1* is a main contributor to the transcriptional regulations in proestrus females. To further investigate the genes and their related biological functions under the direct transcriptional control of *Egr1* in a sex-specific and estrous cycle-specific manner, we analyzed Egr1 targets in the mPFC of males, proestrus, and diestrus females by ChIP-seq.

**Fig. 5:**
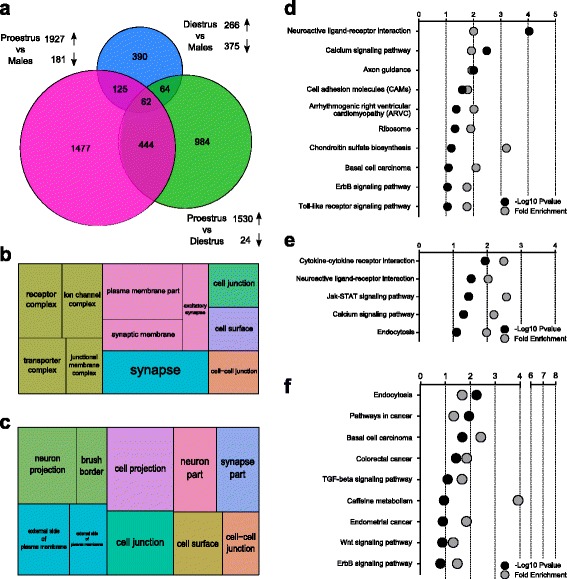
Egr1 binds to synapse-related genes in a sex-specific and estrus cycle-specific manner. **a** The majority of differential Egr1 binding was observed in proestrus when compared to either males (*pink circle*) or diestrus females (*green circle*). **b**, **c** treemap representation of Cellular Compartment terms from the Gene Ontology database, showing a marked enrichment of receptors complexes, synapse, and zones of cell–cell communication localizations in proestrus when compared to males (**b**) or diestrus (**c**). The size of each rectangle is proportional to the -log10 of the *p*-value (the bigger the rectangle, the more significant the enrichment). **d**–**f** The enrichment analysis of pathways from the Kyoto Encyclopedia of Genes and Genomes on the nearest genes to a differential Egr1 binding locations reveals an alteration of signal transduction pathways between males and females in proestrus (**d**) and diestrus (**e**), as well as within females (**f**)

In line with the regulation of *Egr1* mRNA levels specific to proestrus, Egr1 differential binding was predominantly observed between proestrus and males (2108 hits), and within females (1554 hits), whereas it was less notable between diestrus and males (641 hits, Fig. 5a). In proestrus, the nearest located genes were strongly associated with neuronal and, especially, synaptic compartments when compared to males (Fig. 5b) or diestrus (Fig. 5c). Furthermore, a similar modulation of neuronal signal transduction (neuroactive ligand-receptor interaction, calcium signaling pathway, axon guidance, cell adhesion molecules, Jak-STAT signaling pathway, endocytosis) was observed in all comparisons, although seemingly weaker in proestrus versus diestrus (Fig. 5d–f). Altogether, these observations confirmed the involvement of *Egr1* in the transcriptional regulation of the mPFC in proestrus, and highlighted an interesting association of its targets in the regulation of synaptic function.

Finally, to link the sex-specific and estrous cycle-specific differential binding of Egr1 to the transcriptomic variations, we specifically analyzed the genes common in both RNA-seq and ChIP-seq datasets. Only the proestrus versus diestrus comparison showed an overlap of 92 common genes, strongly associated with a wide range of processes related to synaptic function, from synaptic and postsynaptic localizations to synaptic architecture, neurotransmitter transport, recycling, and secretion (Table 3). This denotes a direct control by *Egr1* of the alterations in synaptic functions highlighted by the differential gene expression in the mPFC throughout the estrous cycle.

**Table 3:** Top 3 annotation clusters enriched in proestrus versus diestrus common between RNA-sequencing and Chromatin immunoprecipitation-sequencing analyses

Cluster enrichment score	Term	Count	*p*-value	Fold enrichment
Cluster #1: 2.72	Synapse	14	3.07E−08	7.24
	Cell junction	13	1.27E−06	5.82
	Plasma membrane	29	1.77E−06	2.37
	Plasma membrane part	21	4.06E−06	3.03
	Synapse part	10	4.81E−06	7.56
	Postsynaptic membrane	7	5.92E−05	10.01
	Synaptic transmission	7	5.03E−04	6.78
	Cell-cell signaling	8	7.78E−04	5.14
	Transmission of nerve impulse	7	1.90E−03	5.25
	Exocytosis	5	2.01E−03	9.14
	Postsynaptic density	4	8.24E−03	9.44
	Secretion by cell	5	1.51E−02	5.15
	Neurotransmitter secretion	3	2.41E−02	12.24
	Secretion	5	3.49E−02	3.97
	Vesicle-mediated transport	6	5.15E−02	2.91
	Regulation of neurotransmitter levels	3	5.77E−02	7.58
	Neurotransmitter transport	3	7.82E−02	6.36
	Generation of a signal involved in cell-cell signaling	3	8.78E−02	5.95
	Regulation of cellular localization	4	1.37E−01	3.06
	Protein domain specific binding	4	1.99E−01	2.55
	Regulation of secretion	3	3.14E−01	2.60
	Neurological system process	9	6.40E−01	1.03
Cluster #2: 2.30	Biological adhesion	9	1.23E−03	4.12
	Cell adhesion	9	1.23E−03	4.12
	Cell-cell adhesion	4	8.08E−02	3.87
Cluster #3: 1.74	Postsynaptic membrane	7	5.92E−05	10.01
	Cytoskeleton	11	1.70E−02	2.29
	Cytoskeletal part	9	2.15E−02	2.54
	Intracellular non-membrane-bounded organelle	12	2.99E−01	1.29
	Non-membrane-bounded organelle	12	2.99E−01	1.29

## Discussion

In this study, we found that the sexually biased transcriptome was distinct in proestrus and diestrus females. Surprisingly, while relatively few genes were sexually biased, we observed a profound reorganization of the rat mPFC transcriptome throughout the estrous cycle with 10–14 times more DEG between proestrus and diestrus than between males and females in either estrous cycle stage. While both female groups exhibited alterations in cellular communication when compared to males, proestrus females displayed widespread up-regulation of genes and exon usage related to synaptic neurotransmission, which represented the preponderant sex × estrous cycle alteration. Furthermore, proestrus females showed a specific down-regulation of *Egr1* levels, along with variations in its binding at synaptic function-related genes. This association was particularly strong for the genes being also differentially expressed, revealing a direct involvement of *Egr1* in the specific transcriptomic signature of proestrus.

The advances in genome-wide analyses tools such as microarrays and RNA-seq have allowed a better understanding of sex differences in gene expression in various tissues and organisms. As such, and contrary to somatic tissues such as the liver, adipose, or muscle tissue, a limited sex bias is observed in the brain [16–18, 21]. In the rat mPFC, we found 67 sexually biased genes (0.43 % of all detected genes), in line with the human PFC where the sex bias was only 0.14–0.24 % (representing 22–35 genes, respectively) [17, 18]. Such small differences can appear limited in view of the profound differences in mPFC physiology [6], and could result from the high cell-type heterogeneity within cortical tissues [22]. Nevertheless, given the relatively wide sex bias observed in genes related to protein degradation and translation, an amplification at the protein level cannot be ruled out. Notably, we show that, despite not affecting the number of DEG, the estrous cycle does impact the biological significance of the sexually biased transcriptome. Interestingly, while confirming sex differences in translation, ECM organization, and mitochondrial function described in the mouse and human brain (including PFC) [16, 17, 23], we detailed partly opposite regulations in proestrus and diestrus females when compared to males or to each other. Indeed, interaction with the ECM was higher in proestrus, and mitochondrial function and translation were enriched in diestrus. In line with our observations, such up-regulation of mitochondrial function has already been reported in the rat mPFC, where cytochrome c oxidase activity was greater in diestrus than estrus [24]. It is particularly interesting to note that in addition to the multitude of variations observed within females, the majority of genes were up-regulated in proestrus, but down-regulated in diestrus when compared to males (Fig. 4, cluster 1). As a result, this variability is likely to mask a substantial number of sex differences specific to either stage of the estrous cycle. Variations in synaptic function, for instance, are among such processes whose enrichment was revealed after discriminating between proestrus and diestrus, and illustrate the importance of accounting for the estrous cycle in experimental designs [3].

Given the extent of gene regulation observed within females, we favored an analysis of biological pathways and processes over gene-based or transcript-based investigations in order to capture an integrative understanding of the sex and estrous cycle effects, and thus identified a multi-level up-regulation of processes related to synaptic transmission in proestrus. Accordingly, without consideration of the estrous cycle, female rats display fewer neurons and glia, smaller structure volume, lower spine density, as well as shorter and less branchy apical dendritic arbors than males in the mPFC [25–27]. However, as illustrated by our study, such differences are affected by the estrous cycle and depend on cyclic ovarian hormone fluctuations, with higher spine density in cortical neurons dendrites in proestrus than estrus or diestrus [28, 29]. In other brain regions such as the hippocampus, these regulations are paralleled by variations in synaptic activity and plasticity [10], with male rats generally displaying slightly higher basal activity than females [30, 31]. Within females, however, the estrogen surge in proestrus increases the excitability of CA1 and CA3 pyramidal neurons over other estrous cycle stages, including diestrus [31, 32]. It is interesting to note that such effect is dependent on the slow rise of estrogen the preceding day [33], which thus strengthens the importance of cyclicity in hormonal fluctuations. Although similar regulations have been reported in other regions, the effects of sex and the estrous cycle on the mPFC electrophysiology remain unclear. Gamma-aminobutyric acid (GABA) binding, as well as mPFC contents of serotonin, dopamine, and their respective metabolites, differ between male and female rodents and vary across the estrous cycle [34–36], whereas glutamatergic transmission is higher in proestrus than in diestrus [37]. Through widespread modulations of these systems, ovarian hormones represent the main candidates in mediating such variations [10]. Interestingly, estrogen treatment in ovariectomized rats induces, in the mPFC, a reorganization of genes and pathways common with our study, including neurotransmission, signal transduction, transport, transcription, ECM, and cell adhesion [15]. Furthermore, in our study we found 399 genes (41 % of DEG) with known regulation by estrogens or progestins between proestrus and diestrus, 63 of which (16 %) are related to synaptic function at multiple levels, including synaptic assembly, neurotransmitter release and metabolism, and ion channels, as well as post-synaptic receptors and their downstream signaling (Additional file 14: Table S7; Additional file 15: Table S8). Combined with the enrichment of processes related to neurotransmission at the structural (cell–cell junction, ECM, focal adhesion, actin cytoskeleton), receptor (G protein-coupled receptor signaling, intracellular signaling pathways), and transporter (slc-mediated transmembrane transport, transmission synapse) levels in proestrus, these observations strongly support substantial sex differences in synaptic structure, function, and plasticity governed by hormonal fluctuations across the estrous cycle.

We observed a strong contribution of the neuronal cell type (both “pyramidal neuron,” and “interneuron”) to the profile of gene expression between proestrus and diestrus females (Additional file 11: Figure S6; Additional file 12: Figure S7). In addition to further supporting the preponderance of synaptic transmission in regulation by the estrous cycle (Fig. 4), this finding strengthens existing observations that both excitatory and inhibitory transmissions are affected by the estrous cycle or ovarian hormones [34, 37]. Similarly, the suggested contribution of pericytes, vascular smooth muscle, and, to a lesser extent, endothelial cells to the regulations by sex are in line with the known sex differences in brain morphology as well as the cerebrovascular system and endothelial cell function and reactivity [5, 25–27, 38]. Notably, sex hormones, and particularly estrogen, strongly regulate cerebrovascular and endothelial cell function and reactivity [38–40], and alter the expression of genes related to vascular transport [15], which could thus explain the more pronounced enrichment of the mural and endothelial cell types in the DEG between proestrus and males than between diestrus and males. Similarly, we observed a suggested contribution of oligodendrocytes to the regulations by sex or the estrous cycle, although at different maturation stages, in accordance with the known sex differences in oligodendrocytes number, white matter volume, myelin sheaths, and myelinated fibers in the rodent brain [41]. Given the crucial roles played by each of these cell types in regulating neuronal signal transmission, these observations strengthen evidence for the widespread nature of the regulation of synaptic transmission by sex and the estrous cycle.

Among the genes affected by sex or the estrous cycle, we detected several key transcription factors likely to explain part of the transcriptomic and biological pathway regulations. For instance, it is interesting to note that the transcription factors *Fosb*, *Maff*, *Bcl6b*, and *Klf4*—all down-regulated in females when compared to males—exhibit high expression in endothelial and mural cells in the mouse brain [22, 42], and regulate endothelial cell function and ECM components [43–46] in line with the enrichment of these cell types in the sexually biased gene expression profiles. Furthermore, *Maff* and *Klf4* are also induced in response to nerve-growth factor in rat PC12 cells alongside several other immediate early genes, such as *Egr1*,*2*,*4* or *Id1*, that are all differentially expressed in proestrus females when compared to males (Additional file 2: Table S1), which highlights their involvement in neuronal function. Nevertheless, among these regulations, we detected an interesting alteration of the immediate early gene *Egr1*. Indeed, *Egr1* was down-regulated in females, and was identified as a main contributor to the down-regulation of transcription-related processes between proestrus females and males. Furthermore, *Egr1* was revealed as a main candidate transcription factor associated with sexually biased genes in proestrus females by an in silico analysis (Additional file 13: Table S6), which was further detailed by our ChIP-seq analysis by underlying its particular involvement in the transcriptional signature of proestrus females, with a direct association with synapse-related genes.

Despite its widely accepted involvement in and regulation by synaptic activity, the exact targets of Egr1 and their respective connection to the control of synaptic functions remain unclear [47]. Indeed, in the cortex, *Egr1* is induced by synaptic activity or major signaling factors such as Elk-1, NF-κB, Egr1 itself, or the mitogen-activated protein kinase (MAPK) pathway [47–49]—which, notably, was up-regulated in proestrus. Moreover, although its regulation by ovarian hormones in the mPFC remains to be characterized, *Egr1* is at the center of a gene regulation network induced by estrogen in the mouse mammary gland [50], and while estrogen up-regulates *Egr1* mRNA in the mouse uterus via activation of the MAPK pathway, co-treatment with progesterone dampens this effect [51]. Because tissues used in our study were collected in the early afternoon of proestrus, the down-regulation of *Egr1* mRNA in proestrus may result from the early rise in progesterone levels in this stage of the cycle. Nevertheless, proestrus females exhibited a widespread differential binding of Egr1 to its transcriptional targets when compared to males or diestrus females, suggesting enhanced Egr1-mediated transcriptional regulations despite lower mRNA levels. We could thus identify a proestrus-specific alteration of transcriptional regulators including *Egr1* itself and some of its targets previously associated, in different systems, with pathways and processes regulated by the estrous cycle in the rat mPFC. Indeed, *Egr1* regulates ECM composition, mitochondrial function, apoptotic processes, signal transduction, and transcription, through the transcriptional control of a variety of genes [52–54], of which several are differentially expressed in proestrus. Furthermore, in vitro evidence for a role of *Egr1* in the control of synaptic functions in neurons exist, as its overexpression in rat PC12 cells affects the expression of 135 genes—the majority being down-regulated—related to synaptic function, including neurotransmitters, signal transduction, presynaptic vesicular trafficking, synapse formation and assembly, and protein translation and degradation [54]. In our study, we report a similar enrichment from the genes showing a differential Egr1 binding in proestrus, especially among DEG (Table 3). Combined to the over-representation of *Egr1* among the transcription factors associated with the gene expression profile of proestrus females (Additional file 13: Table S6), these data support a direct control of synapse-related genes by Egr1 throughout the estrous cycle in the rat mPFC. It is important to note, however, that the contribution of Egr1 is likely not exclusive because Egr1 can form heterodimers with other transcription factors involved in neuronal function and activity, such as Fos or Jun [47, 55, 56]. In addition to extending the range of Egr1 targets, this highlights an additional layer of complexity in transcriptional regulations by sex and the estrous cycle.

Alongside synaptic changes, oxidative phosphorylation and ribosome-associated genes were enriched in females, in line with previous transcriptomic and enzymatic observations [16, 17, 23]. While both these processes essential to synaptic plasticity vary throughout the estrous cycle and are regulated by ovarian hormones in various systems [24, 57–61], we revealed a diestrus-specific up-regulation of their related genes in the mPFC. It is important to note, however, that this suggested down-regulation of mitochondrial function and translation in proestrus appears in contradiction with the classically reported enhancing effects of estrogen [61]. Nevertheless, while estrogen and progesterone both stimulate mitochondrial function when analyzed separately, progestins can antagonize estrogens’ effects in the female rat brain [61], and extensively down-regulate ribosome-associated genes in other systems [59, 60]. This down-regulation in proestrus could thus result from the early rise in progesterone at this stage of the cycle. Similarly, the enhancing effects of estrogens—rising in the morning of proestrus—on these processes are thought to represent rapid non-genomic effects [62], which could therefore be on the decline in the afternoon of proestrus. Nevertheless, genomic and non-genomic effects of sex hormones are not exclusive but may rather act in concert [63] and result in complex transcriptomic variations throughout the estrous cycle. Non-genomic effects, for instance, were proposed to initiate a rapid enhancement of synaptic plasticity through MAPK-dependent and Akt-dependent signaling and actin cytoskeleton remodeling that would be further stabilized in the event of sustained synaptic activity [62]. Because MAPK, Akt, and actin remodeling pathways are enriched in proestrus, it is tempting to hypothesize that their gene expression profile would thus prepare the female mPFC for the hormonal surge in proestrus. Interestingly, following a small-scale study of protein expression by western blotting, we could confirm the profiles of regulations of the majority of targets assessed (Additional file 3: Figure S2c, d), suggesting that the alteration of translation-related processes observed between sexes and estrous cycle stages represents an additional component of the regulation of synaptic transmission, as previously suggested [57]. Nevertheless, we cannot rule out the existence of compensatory mechanisms at the protein level affecting different targets or biological pathways.

Females in proestrus thus exhibit a widespread transcriptomic reorganization, partially under the control of Egr1, that suggests differences in synaptic activity in the mPFC when compared to either males or diestrus females. Because the integration of afferent signals by the mPFC is critical in controlling both perception of the environment and the corresponding behavioral response [7], these transcriptomic variations could underlie sex and estrous cycle differences in perception and response to anxiogenic environments. Interestingly, sex-dependent and estrous cycle-dependent variations in anxiety levels and perception of aversive elements are reported in both women and female rodents, although are variable between strains and experimental paradigms [9, 10, 64]. In addition, estrogen, progesterone, and their metabolites can alter anxiety levels through modulation of dopaminergic, serotoninergic, and GABAergic systems [10], whose mRNA levels vary between proestrus and diestrus. Interestingly, in premenstrual dysphoric disorder, anxiety and depressed mood occur around the onset of menstruation and present with different sensitivities of serotonin and GABA receptors [10]. Furthermore, our transcriptomic regulations overlap with those recently identified as critical regulators of anxiety behaviors in the male mouse mPFC and several neuropsychiatric disorders [65], supporting the association of our DEG with depression, behavioral diseases, and bipolar disorders (Additional file 7: Figure S4). Notably, alterations in genes related to synaptic assembly and transmission, cell communication, mitochondrial function, protein translation and degradation, or neurotransmitter systems are recurring features reported in a variety of brain regions upon cognitive decline or neuropsychiatric disorders [66–69], with which Egr1 is associated [66, 67]. Altogether, these clinical and pre-clinical data suggest a critical role for transcriptomic regulations in the adult rat mPFC by the estrous cycle in modulating the organism’s interaction and perception of its environment.

## Conclusions

Sex differences are prominent in cognitive functions and neuropsychiatric disorders. However, despite the known influence of ovarian hormone fluctuations on these processes in females, surprisingly little is known regarding the underlying mechanisms. Here, we showed that the extent of transcriptomic regulation throughout the estrous cycle vastly exceeds sex differences, and critically affect the sexually biased biological functions. Indeed, accounting for the estrous cycle by investigating females in a state of high or low levels of ovarian hormones—proestrus or diestrus, respectively—revealed the existence of estrous cycle-specific sex differences. As such, the preponderant regulations, supported by 64.7 % of all DEG, corresponded to a proestrus-specific enrichment of synapse-related genes, partly under the direct transcriptional control of the immediate early gene *Egr1*, a known mediator of sex differences in anxiety-like behaviors. Females in diestrus, on the other hand, exhibited a specific enrichment of translation and mitochondrial function-related genes. In addition to illustrating the critical influence of the estrous cycle on the rat mPFC phenotype and its interaction with sex differences, our transcriptomic investigation identified sex and estrous cycle regulations, providing the groundwork for a better understanding of the female specificities in cognition and mood regulation.

## Methods

### Subjects

Eight-week-old male and female Sprague Dawley rats (Charles River Laboratories, Wilmington, MA, USA), weighing 250–275 g or 200–225 g, respectively, were used in this study. Males and females were randomly pair-housed in separate rooms and maintained on a 12 h light/dark cycle (lights off at 19:00) with food and water available ad libitum. All manipulations were performed in accordance with the guidelines of the Animal Care and Use Committee of Florida State University and National Institutes of Health guidelines.

### Determination of estrous cycle in females and tissue collection

Following 5 days of habituation and handling under pair-housing, the estrous cycle of female rats was assessed daily by vaginal smearing and subsequent cytological analysis [70] for a minimum of two cycles in order to ensure proper and regular cyclicity. When both animals of the same pair were at the desired stage of the estrous cycle, both animals were killed and their brain quickly dissected out, snap-frozen, and stored at −80 °C until further processing. To account for eventual variability caused by differences in day of tissue collection, male rats were killed the same day as females. As one of the aims of this study was to investigate estrous cycle-dependent effects, we chose to compare stages of low and high levels of sex hormones. Females were thus killed either early in the first day of diestrus (low hormonal levels) or in the early afternoon of proestrus (high hormonal levels), when the estrogen peak is still pronounced and progesterone levels are rising [70, 71]. To reduce variability between samples, only females with regular, 4-day cycles were considered. Of note, separate batches of animals were used for RNA-seq and ChIP-seq studies.

### RNA extraction, library preparation, and sequencing

Total RNA was extracted from mPFC tissue punches (n = 5 per group) containing the infralimbic, prelimbic, and cingulate cortices using the TRI-Reagent protocol according to the manufacturer’s instructions (Molecular Research Center, Cincinnati, OH, USA ) followed by DNAse I treatment to remove any eventual DNA contamination and clean-up (RNA Clean & Concentrator, Zymo Research, Irvine, CA, USA). The RNA integrity was then assessed electrophoretically on an RNA StdSens Experion chip (Bio-Rad, Hercules, CA, USA), which reported that all samples had an RNA quality indicator number (RQI) ≥ 8.8. Four biological replicates from each group were then selected for further processing based on the consistency of their estrous cyclicity, highest RQI, and concentration homogeneous to other samples.

RNA-seq libraries were prepared using the NEBNext Ultra RNA Library Prep Kit for Illumina with poly(A) mRNA purification from 1 μg of total RNA based on magnetic beads, cDNA synthesis using random hexamers, and final amplification using barcoded primers following the manufacturer’s protocol (#E7530, New England Biolabs, Ipswich, MA, USA). To determine the lower limit of detection and verify the linearity of quantification during the statistical analysis of the sequencing data, synthetic RNA Spike-Ins (#4456739, ERCC ExFold RNA Spike-In Mixes, Life Technologies, Carlsbad, CA, USA) were added to each sample prior to poly(A) mRNA purification following the recommended dilutions (2 μL of a 1:100 dilution). Of note, Mix 1 and Mix 2 of the ERCC ExFold RNA Spike-Ins were equally distributed among samples in an exclusive manner. The resulting barcoded and unstranded libraries were quantified using a KAPA qPCR library quantification kit (KAPA Biosystems, Boston, MA, USA) with three serial dilutions ran in duplicate on a CFX96 real-time PCR instrument (Bio-Rad). Finally, the absence of adapter or primer contamination was verified on a Bioanalyzer using a DNA High Sensitivity chip (Agilent Technologies, Santa Clara, CA, USA).

To maximize sequencing depth while avoiding lane-specific bias during sequencing, all 12 barcoded libraries (four biological replicates per group) were pooled before being sent to the Interdisciplinary Center for Biotechnology Research at the University of Florida for sequencing on a NextSeq 500 (Illumina, San Diego, CA, USA) in High-Output 1 × 150 bp mode. This design allowed the generation of 415.96 M single-end raw reads (passing filter, >Q30, and demultiplexed), with a median number of reads per biological sample of 34.22 M.

#### Chromatin immunoprecipitation, ChIP-seq library preparation, and sequencing

ChIP was performed as previously described [72], with slight modifications. Briefly, cross-linked chromatin was sheared to fragments of 200–500 bp using a Bioruptor sonicator (Diagenode, Denville, NJ, USA). Pre-cleared chromatin was then immunoprecipitated overnight at 4 °C with an antibody directed against Egr1 (sc-110-X, 4 μg, Santa Cruz Biotechnology, Dallas, TX, USA). After washing, elution from beads, and reversal of the cross-linking, immunoprecipitated DNA was purified and the specific enrichment versus DNA immunoprecipitated with normal rabbit IgG (EMD Millipore, Billerica, MA, USA) was assessed by real-time PCR.

A total of 11 samples were then used for the generation of ChIP-seq libraries using the NEBNext Ultra DNA Library Prep Kit for Illumina and barcoded primers (New England Biolabs) following the manufacturer’s protocol (#E7370). For each biological sample, two libraries were prepared (IP and Input), resulting in a total of 22 barcoded libraries. All libraries were quantified and verified for the absence of adapter or primer contamination as described above.

To maximize the depth of sequencing per sample despite the high number of libraries, samples were pooled in four separate tubes while ensuring an equal distribution of biological condition across tubes and avoiding separation of IP and corresponding Input libraries. Tubes 1–3 contained one male, one proestrus female, and one diestrus female each (corresponding to a total of three IP and three matching Input libraries per tube), whereas tube 4 contained two diestrus females. Each pool of libraries was once again quantified and verified for the absence of adapter or primer contamination before being sent to the Translational Science Laboratory at Florida State University for sequencing on an HiSeq2500 (Illumina) in rapid-run 1 × 100 bp mode. This design generated a total 528.96 M single-end raw reads (passing filter, >Q30, and demultiplexed), with a median number of reads per individual library of 21.31 M.

### Processing of sequencing data

For RNA-seq data analysis, raw reads were first processed for quality filtering and adapter trimming with Trimmomatic [73]. Following a final verification of good quality by FastQC [74], each library was mapped against the rat genome and annotation from Ensembl release 76 (Rnor_5.0) using Tophat2 (v2.0.11) [75] and the --b2-very-sensitive Bowtie2 (v2.1.0) preset. Reads were aligned to the rat reference genome to which the ERCC spike-ins sequences were added, as a result of a prior evaluation of the linearity of the spike-ins detection following either simultaneous or sequential mapping to each reference separately in either order (rat genome or ERCC spike-ins), which revealed that combining both references during the mapping step provided the best linearity, lower limit of detection, and dynamic range (see below). The number of reads mapping uniquely to each gene was counted by HTSeq-count [76] and processed for statistical analysis using the R Bioconductor packages edgeR (v3.6.7) and DESeq2 (v1.4.5) [77–79]. To limit the number of false-positives throughout the analysis, only the genes detected as differentially expressed with an FDR of 5 % using both statistical packages were retained. Prior to proceeding to the differential expression analysis of reads mapping to the rat genome, reads mapped uniquely to ERCC spikes-ins were processed through both the edgeR and DESeq2 analysis pipeline to assess the lower limit of detection, from which the minimum number of reads for an accurate measure of gene expression was calculated. Genes that did not satisfy this minimum number of uniquely mapped reads were discarded from the dataset before statistical analysis following each package’s recommendations. In addition, early data analysis and visualization revealed the presence of one outlier in the Proestrus group that was then removed from subsequent analyses, as recommended by edgeR’s manual. As previously observed, the number of DEG and their respective fold-change detected by the edgeR package were slightly greater than that of the DESeq2 package (Additional file 16: Figure S8). In an effort to limit false-positives and use more stringent conditions, the fold-change values from the DESeq2 package were used when necessary (heatmap generation and cluster analysis). In addition, differences in splicing events, alternative transcription start sites, and polyadenylation site usage were investigated using the R package DEXSeq (v1.10.8) [20], with a more stringent FDR threshold of 1 % to account for the increased number of comparisons.

For ChIP-seq data analysis, raw reads were processed for quality filtering and adapter trimming as described above. Each library was then mapped to the Rnor_5.0 genome (the same used for our RNA-seq data) using Bowtie2, and the uniquely mapping reads with a mapping quality superior or equal to 30 were conserved. After removal of duplicates, all libraries were processed through the Irreproducibility Discovery Rate pipeline [80–82] to identify and remove outliers, resulting in a final number of three males, two proestrus females, and four diestrus females replicates. Differential ChIP-seq signal was then analyzed using the DiffReps [83] tool for each pairwise comparison, proestrus versus males, diestrus versus males, and proestrus versus diestrus, and annotated with the R package ChIPpeakAnno (v2.14.1) [84].

#### Functional analysis

To identify distinct patterns of regulation between males, proestrus females, and diestrus females, genes were clustered based on the profile of regulation of their raw counts across all conditions using the MultiExperiment Viewer software [85]. After median centering, the optimal number of clusters was determined by figure of merit plot [86], and K-means clustering was performed using the Euclidian distance as the metric.

Functional enrichments of annotations from the GO consortium, Disease Ontology, or the KEGG were computed, analyzed, and visualized using the Database for Annotation, Visualization and Integrated Discovery (DAVID, v6.7) [87], DOSE [88], GOrilla [89], and ReviGO [90] tools. In addition, GSEAs [91] were performed using gene sets comprising pathway annotations for rat curated from public databases (http://download.baderlab.org, November_06_2014 release), and the resulting enriched pathways were visualized using the Cytoscape (v3.2.0) enrichment map plugin [92], following the author’s recommendations. The analysis of *Egr1* enrichment in our list of DEG was conducted using enrichR [93], which computed the over-representation of transcription factors from the ChIP Enrichment Analysis database [94].

The regulation of genes by estrogens and progestins was analyzed by comparing our list of DEG with those that were previously identified as being regulated by estrogens or progestins in the Comparative Toxicogenomics Database [95] as of 2 March 2015. DEG with a previously reported regulation by either estrogens or progestins were extracted, and their functional enrichment was analyzed using the functional clustering tool from DAVID [87].

Similarly, the contribution of different cell types to the profiles of gene regulations we observed was analyzed by comparing our dataset to two different publically available RNA-seq datasets analyzing gene expression in different mouse brain cell types. The first dataset corresponds to a single-cell RNA-seq dataset [22] performed on young (P20–P31) mouse brains (male and female) investigating the following cell types: astrocytes, vascular endothelial cells, ependymal cells, interneurons, microglia, mural cells (pericytes and vascular smooth muscle cells), pyramidal neurons (hippocampal CA1 and somatosensory S1 pyramidal cells were combined for our analysis), and oligodendrocytes. The second dataset [42] originates from sequencing of fluorescence-activated cell-sorted tissue from cerebral cortices of juvenile mice (P7 and P17) analyzing the following cell types: astrocytes, endothelial cells, microglia, neurons, oligodendrocyte precursor cells, newly formed oligodendrocytes, and myelinating oligodendrocytes. For each of these datasets, each gene was attributed a cell type (the one with highest expression), and the enrichment of each cell type in our lists of DEG (FDR 5 %) was then tested using a one-sided Fisher’s exact test. DEG in each pairwise comparison from each significantly enriched cell type were then extracted and processed for functional annotation analysis using the GO and KEGG databases by DAVID [87].

### Analysis of mRNA levels by real-time PCR

One microgram of total RNA from the same samples used for RNA-seq library preparation was analyzed by semi-quantitative real-time PCR as previously described [96], with normalization to hypoxanthine phosphoribosyltransferase 1 gene, which was not differentially expressed between males and females in our RNA-seq data (DESeq2 FDR = 0.999). All reactions were performed in triplicate on a CFX384 thermocycler (Bio-Rad), and their specificity was verified by melting curve analysis. All primers used are detailed in Additional file 17: Table S9. Normalized data were analyzed using the StatView software (v5.0.1, SAS Institute) by one-way analysis of variance followed by Tukey’s post-hoc test when a main effect was statistically significant.

### Analysis of protein levels by western blotting

To confirm whether the transcriptomic changes would carry at the protein level, the expression levels of nine proteins selected based on their relevance to the biological pathways affected by sex or the estrous cycle were measured on the same samples used for RNA-seq by western blotting as previously described [72]. Briefly, proteins were transferred to nitrocellulose membranes following separation on 12 % sodium dodecyl sulfate polyacrylamide gels, and incubated with the following primary antibodies: Tsc1 (#4906), Dnmt3a (#3598), Gad2 (#3988), and Akt (#9272) purchased from Cell Signaling Technology (Danvers, MA, USA); Syt1 (sc-7753), Rgs9 (sc-8143), Igfbp2 (sc-6002), and Igf2 (sc-1415) purchased from Santa Cruz Biotechnology; B3galt1 (ab82760) purchased from Abcam (Cambridge, MA, USA); and Actin (MAB1501) purchased from Millipore (Billerica, MA, USA). After incubation with the corresponding secondary antibodies (LI-COR Biosciences, Lincoln, NE, USA), the membranes were visualized using an Odyssey infrared imaging system (LI-COR Biosciences). The signal for each target was quantified using ImageJ (1.49v, NIH) and normalized to the actin signal, before being analyzed using the StatView software (v5.0.1, SAS Institute) by one-way analysis of variance.

### Availability of datasets

The datasets supporting the results of this article are available in the Gene Expression Omnibus (GEO) repository, under the SuperSeries GSE69773.

## Additional files

Additional file 1: Figure S1. Quantitative characterization of gene expression in the mPFC of males, proestrus, and diestrus females. In (a-d), the raw -log10 of the *p-*values are plotted against the log2 of the fold-change (volcano plot), and the DEG at 5 % FDR are highlighted in red. In (e), the density plot representation of the log2 fold-change of the DEG at 5 % FDR denote the clear proportion of genes up-regulated in proestrus when compared to diestrus, and down-regulated in females when compared to males . In (f), the box-plot representation of the absolute of the log2 fold-change illustrates the relative similarity in the intensity of regulations between all groups. *FvM* females vs males, *PvM* proestrus vs males, *DvM* diestrus vs males, and *PvD* proestrus vs diestrus. In (a-f), values from the R package DESeq2 were used. (PNG 665 kb) Additional file 2: Table S1. Description of the differential expression analysis for all genes detected in the study and for each pairwise comparison. All values correspond to DESeq2 computations. The last column is.common.DE details whether the gene was found differentially expressed (DE) in both edgeR and DESeq2 analyses. FvM: females vs males, PvM: proestrus vs males, DvM: diestrus vs males, PvD: proestrus vs diestrus, padj: false discovery rate. (CSV 4867 kb) Additional file 3: Figure S2. mRNA and protein expression levels of selected genes differentially expressed between males and females by real-time PCR. In (a), the up-regulation in females when compared to males of all four genes selected confirmed the absence of estrous cycle interaction, supporting the accuracy of our RNA-seq analysis. Similarly in (b), the down-regulation of all five genes selected was confirmed. For *Egr1*, a specific down-regulation in proestrus when compared to either males or diestrus was also observed. In (c), two (Rgs9 and Igfbp2) of the three proteins analyzed by western blotting confirmed the sex-biased expression revealed by RNA-seq, whereas in (d) four of the six proteins analyzed exhibit similar trends of proestrus-specific regulations than those observed by RNA-seq. In (c, d), numbers above lines represent *p-*values. All statistical analyses and values are listed in Additional file 4: Table S2. Data are presented as mean ± SEM, and n = 4–5 per group. *M* males, *P* proestrus, *D* diestrus. ^*^ *p* < 0.05, ^**^ *p* < 0.01, and ^***^ *p* < 0.001 vs males; and ^##^ *p* < 0.01 vs proestrus, Tukey’s test. (PDF 145 kb) Additional file 4: Table S2. Summary of all statistical values for the real-time PCR and western blotting data presented in Additional file 3: Figure S2. (XLSX 11 kb) Additional file 5: Figure S3. Association with DNA transcription-related processes of the proestrus-specific sexually biased genes. Association matrix depicting positive (blue) or undocumented (grey) association between each gene (row) and each GO term (column) enriched in the top annotation cluster (Table 2). Note the positive association of *Egr1* with all enriched GO terms related to DNA transcription. (PDF 323 kb) Additional file 6: Table S3. Summary of the functional annotation clustering analysis in each pairwise comparison. (XLSX 149 kb) Additional file 7: Figure S4. Disease Ontology (DO) enrichment analysis on DEG in each pairwise comparison. On the left side is plotted the -log10 of the *p*-value for each DO term significantly enriched, while network graphs depicting each DO term and its associated genes are located on the right side. Note the enrichment of mood-related disorders in the DEG between proestrus and diestrus (a), but not in any of the other pairwise comparisons (b–d). (PDF 194 kb) Additional file 8: Table S4. Summary of the differential exon usage analysis for each pairwise comparison. FvM: females vs males, PvM: proestrus vs males, DvM: diestrus vs males, PvD: proestrus vs diestrus. (XLSX 36690 kb) Additional file 9: Figure S5. Sex-cycle and estrous-cycle bias in exon usage in the rat mPFC. (a) Extensive changes in exon usage are observed between proestrus and diestrus, while only moderate differences were detected between males and females in either estrous cycle stage. (b) Representation of the log2 fold-change over the averaged normalized read counts for the proestrus versus diestrus comparison. In proestrus, 68 % of the differentially expressed features significant at the FDR 5 % threshold (highlighted in red) are up-regulated. (c) The enrichment analysis of pathways from the Kyoto Encyclopedia of Genes and Genomes suggests variations in RNA processing, cell–cell interaction, and protein degradation between proestrus and diestrus. (d) treemap representation of Cellular Compartment terms from the GO database, showing a marked enrichment of nuclear, neuronal, synaptic, and cell–cell contact localizations between proestrus and diestrus. The size of each rectangle is proportional to the -log10 of the *p*-value (the bigger the rectangle, the more significant the enrichment). (PDF 520 kb) Additional file 10: Table S5. Report of the full results of the functional annotation clustering analysis performed on each gene expression clusters described in Fig. 4. (XLSX 29 kb) Additional file 11: Figure S6. Cell type enrichment analysis using the Zeisel *et al.*, 2015 dataset. The contribution of each cell type, as defined by the Zeisel *et al.*, 2015 dataset [22], to the profiles of regulations by sex and the estrous cycle was analyzed by testing the enrichment of each term (cell type) in each pairwise comparison. In (a), the matrix depicts the *p-*value resulting from a one-sided Fisher’s exact test and the background of each cell of the matrix is colored according to the -log10 of this *p-*value. In (b–e), the corresponding genes for each significantly enriched cell type and pairwise comparison were further analyzed for GO and KEGG database using DAVID. (PDF 354 kb) Additional file 12: Figure S7. Cell type enrichment analysis using the Zhang *et al.*, 2014 dataset. The contribution of each cell type, as defined by the Zhang *et al.*, 2014 dataset [42], to the profiles of regulations by sex and the estrous cycle was analyzed by testing the enrichment of each term (cell type) in each pairwise comparison. In (a), the matrix depicts the *p*-value resulting from a one-sided Fisher’s exact test and the background of each cell of the matrix is colored according to the -log10 of this *p*-value. In (b–f), the corresponding genes for each significantly enriched cell type and pairwise comparison were further analyzed for GO and KEGG database using DAVID. (PDF 388 kb) Additional file 13: Table S6. Report of the full results of the transcription factor enrichment analysis performed using the enrichR tool on the DEG in each pairwise comparison. (XLSX 117 kb) Additional file 14: Table S7. Report of the full results of the functional annotation clustering analysis performed on the genes differentially expressed in proestrus vs diestrus with a known regulation by estrogens or progestins. (XLSX 62 kb) Additional file 15: Table S8. Description of the reported interaction of estrogens or progestins on synapse-related genes differentially expressed throughout the estrous cycle in our study. (XLSX 35 kb) Additional file 16: Figure S8. Comparison of differential expression results from the edgeR and DESeq2 R packages. The log2 of the fold-change (a, c, e, g) and -log10 of the FDR values (b, d, f, h) for each pairwise comparison, females vs males (a, b), proestrus vs males (c, d), diestrus vs males (e, f), and proestrus vs diestrus (g, h) for DESeq2 were plotted against the values obtained from edgeR. In all panels, the red dotted line corresponds to a perfect match between the two packages outputs, while the black dotted lines in (b, d, f, h) correspond to the 5 % FDR threshold. In (a, c, e, g), the majority of fold-changes computed by edgeR were of greater amplitude than those of DESeq2, while in (b, d, f, h), the FDR values computed by DESeq2 tended to exceed those from edgeR, highlighting DESeq2 as more conservative than edgeR on our dataset. (PDF 528 kb) Additional file 17: Table S9. Description of all the PCR primers u*s*ed in this study [97–104]. (XLSX 11 kb)

## Abbreviations

ChIP-seq: chromatin immunoprecipitation followed by deep sequencing
DEG: differentially expressed genes
ECM: extracellular matrix
FDR: false discovery rate
GABA: gamma-aminobutyric acid
GO: Gene Ontology
GSEA: gene-set enrichment analysis
KEGG: Kyoto Encyclopedia of Genes and Genomes
MAPK: mitogen-activated protein kinase
mPFC: medial prefrontal cortex
RQI: RNA quality indicator number
