# Supplementary figures and images for: The estrous cycle surpasses sex differences in regulating the transcriptome in the rat medial prefrontal cortex and reveals an underlying role of early growth response 1
Source: Genome Biol. 2015 Dec 2;16:256. doi: 10.1186/s13059-015-0815-x (PMC4667491; doi:10.1186/s13059-015-0815-x)

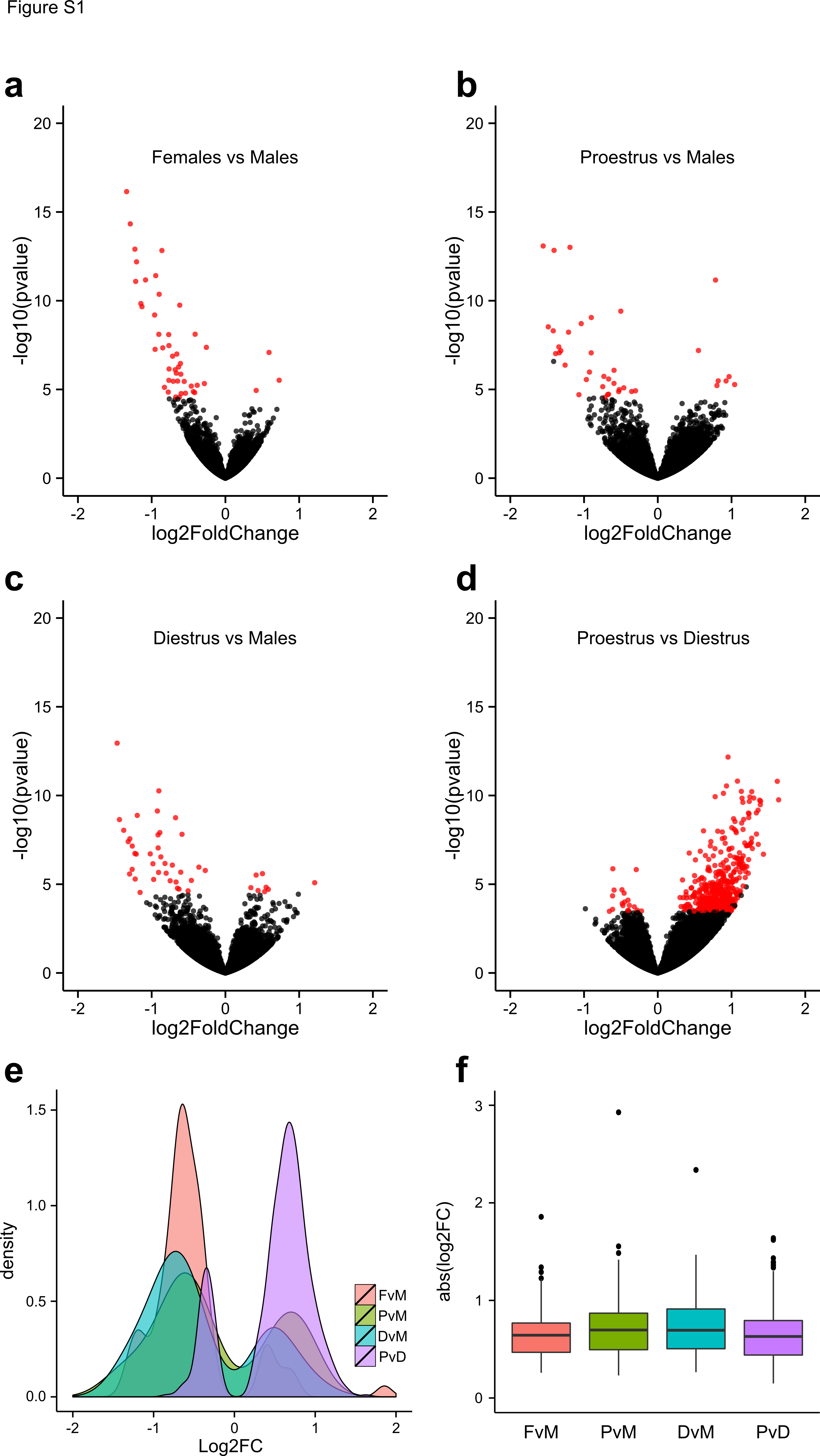

Supplement: Additional file 1: Figure S1. — Quantitative characterization of gene expression in the mPFC of males, proestrus, and diestrus females. In (a-d), the raw -log10 of the p-values are plotted against the log2 of the fold-change (volcano plot), and the DEG at 5 % FDR are highlighted in red. In (e), the density plot representation of the log2 fold-change of the DEG at 5 % FDR denote the clear proportion of genes up-regulated in proestrus when compared to diestrus, and down-regulated in females when compared to males . In (f), the box-plot representation of the absolute of the log2 fold-change illustrates the relative similarity in the intensity of regulations between all groups. FvM females vs males, PvM proestrus vs males, DvM diestrus vs males, and PvD proestrus vs diestrus. In (a-f), values from the R package DESeq2 were used. (PNG 665 kb) [file 13059_2015_815_MOESM1_ESM.png]

Figure S2

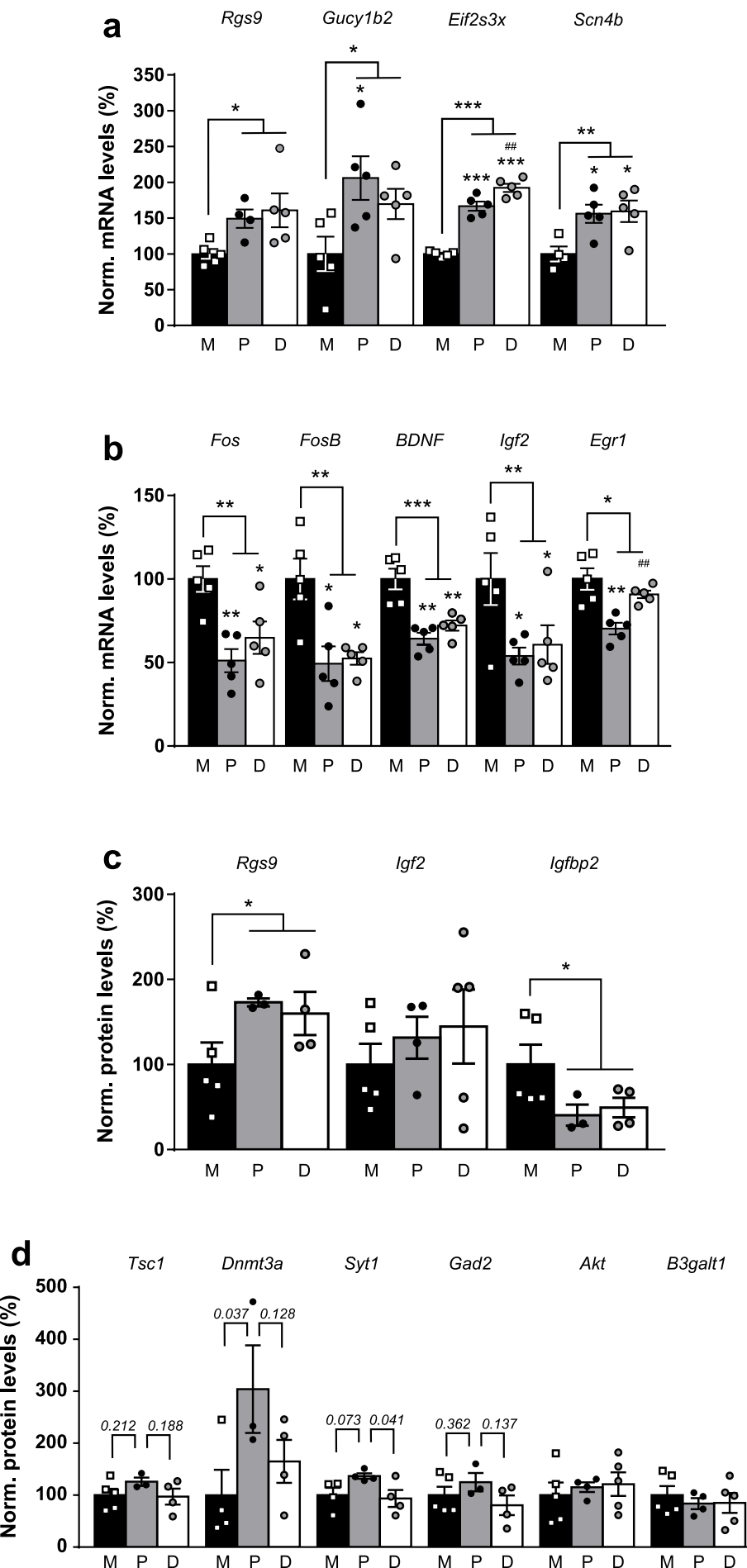

Supplement: Additional file 3: Figure S2. — mRNA and protein expression levels of selected genes differentially expressed between males and females by real-time PCR. In (a), the up-regulation in females when compared to males of all four genes selected confirmed the absence of estrous cycle interaction, supporting the accuracy of our RNA-seq analysis. Similarly in (b), the down-regulation of all five genes selected was confirmed. For Egr1, a specific down-regulation in proestrus when compared to either males or diestrus was also observed. In (c), two (Rgs9 and Igfbp2) of the three proteins analyzed by western blotting confirmed the sex-biased expression revealed by RNA-seq, whereas in (d) four of the six proteins analyzed exhibit similar trends of proestrus-specific regulations than those observed by RNA-seq. In (c, d), numbers above lines represent p-values. All statistical analyses and values are listed in Additional file 4: Table S2. Data are presented as mean ± SEM, and n = 4–5 per group. M males, P proestrus, D diestrus. * p < 0.05, ** p < 0.01, and *** p < 0.001 vs males; and ## p < 0.01 vs proestrus, Tukey’s test. (PDF 145 kb) [file 13059_2015_815_MOESM3_ESM.pdf]

Figure S3

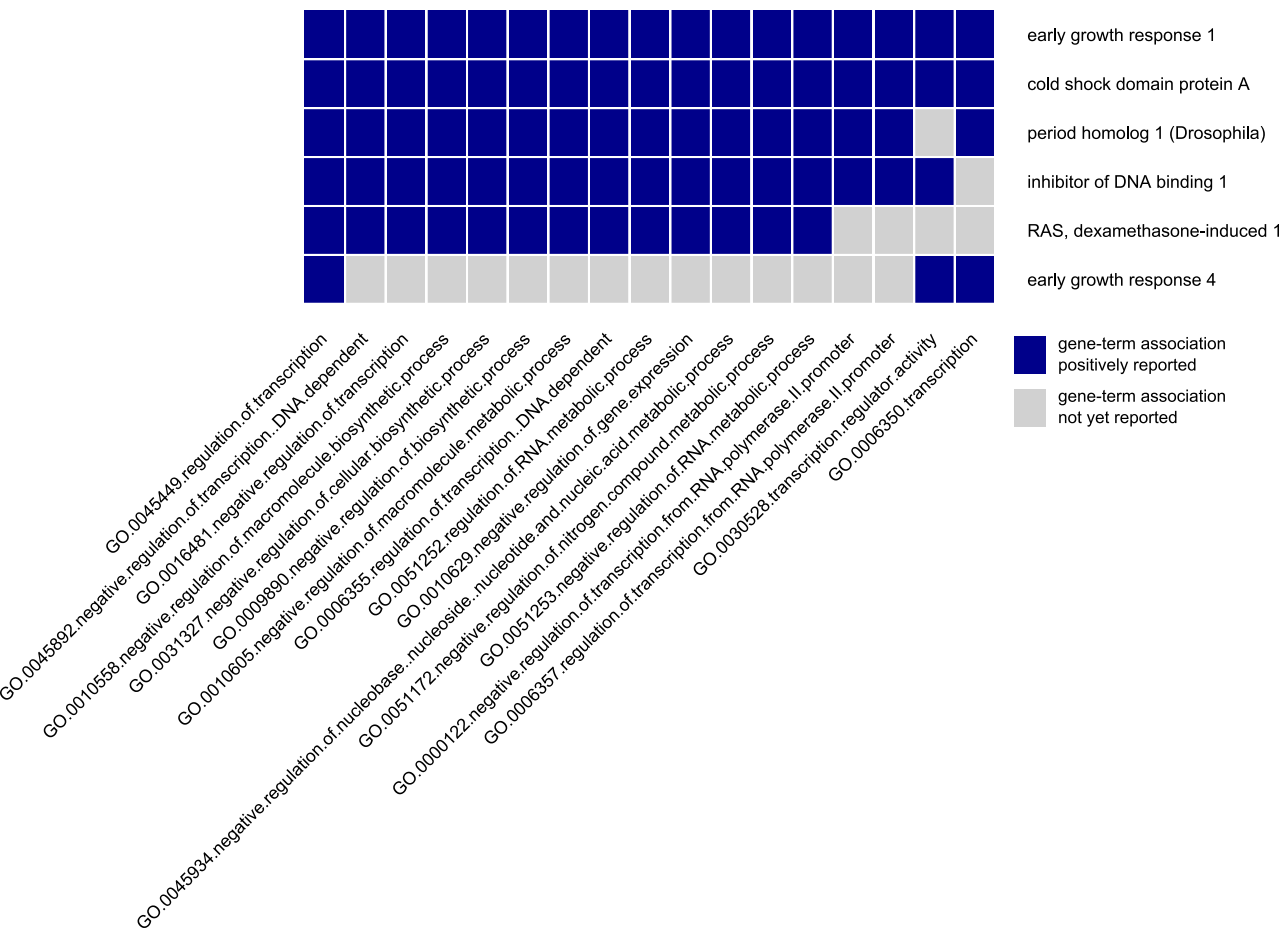

Supplement: Additional file 5: Figure S3. — Association with DNA transcription-related processes of the proestrus-specific sexually biased genes. Association matrix depicting positive (blue) or undocumented (grey) association between each gene (row) and each GO term (column) enriched in the top annotation cluster (Table 2). Note the positive association of Egr1 with all enriched GO terms related to DNA transcription. (PDF 323 kb) [file 13059_2015_815_MOESM5_ESM.pdf]

Figure S4

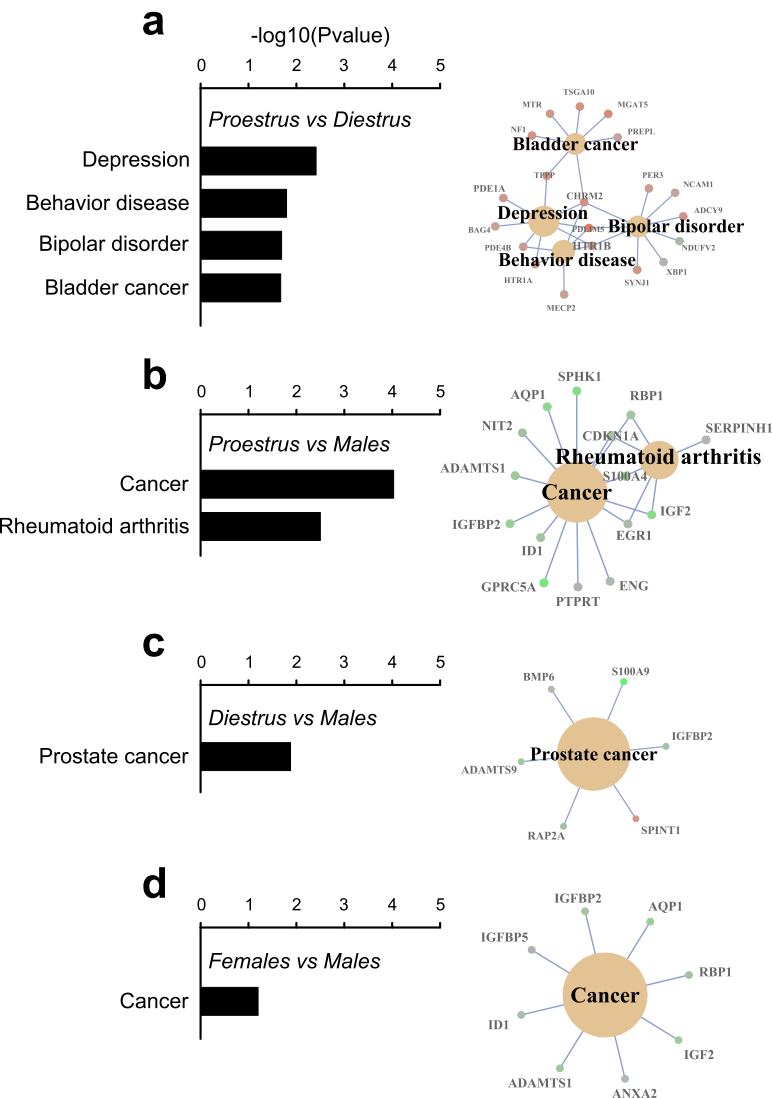

Supplement: Additional file 7: Figure S4. — Disease Ontology (DO) enrichment analysis on DEG in each pairwise comparison. On the left side is plotted the -log10 of the p-value for each DO term significantly enriched, while network graphs depicting each DO term and its associated genes are located on the right side. Note the enrichment of mood-related disorders in the DEG between proestrus and diestrus (a), but not in any of the other pairwise comparisons (b–d). (PDF 194 kb) [file 13059_2015_815_MOESM7_ESM.pdf]

Figure S5

**a**

Proestrus  
vs  
Diestrus

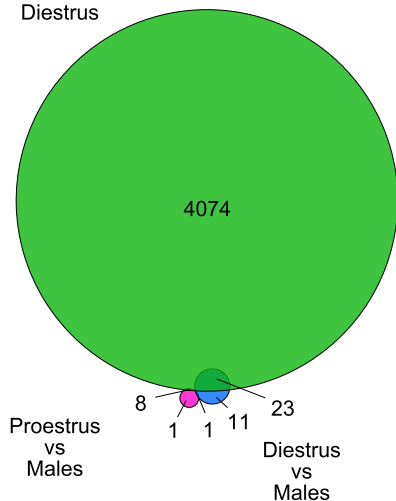**b**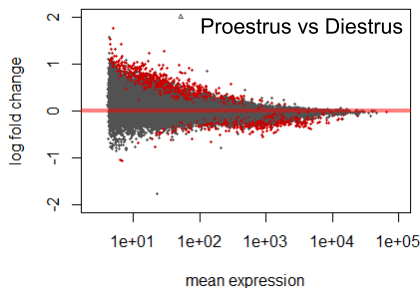**c**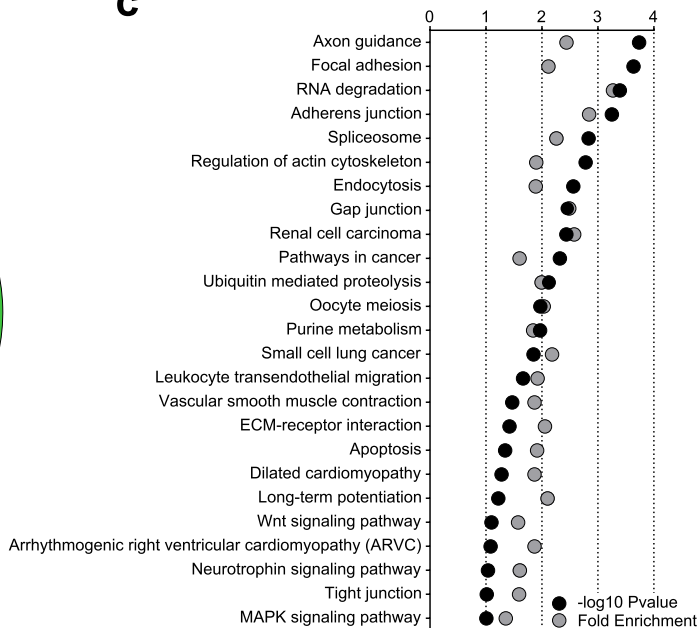**d**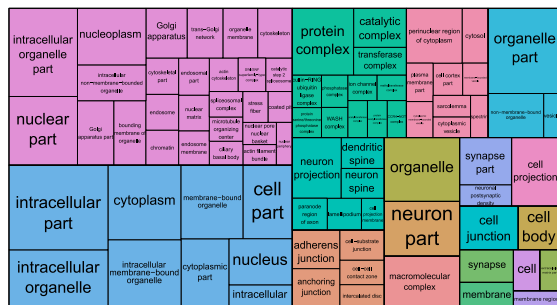

Supplement: Additional file 9: Figure S5. — Sex-cycle and estrous-cycle bias in exon usage in the rat mPFC. (a) Extensive changes in exon usage are observed between proestrus and diestrus, while only moderate differences were detected between males and females in either estrous cycle stage. (b) Representation of the log2 fold-change over the averaged normalized read counts for the proestrus versus diestrus comparison. In proestrus, 68 % of the differentially expressed features significant at the FDR 5 % threshold (highlighted in red) are up-regulated. (c) The enrichment analysis of pathways from the Kyoto Encyclopedia of Genes and Genomes suggests variations in RNA processing, cell–cell interaction, and protein degradation between proestrus and diestrus. (d) treemap representation of Cellular Compartment terms from the GO database, showing a marked enrichment of nuclear, neuronal, synaptic, and cell–cell contact localizations between proestrus and diestrus. The size of each rectangle is proportional to the -log10 of the p-value (the bigger the rectangle, the more significant the enrichment). (PDF 520 kb) [file 13059_2015_815_MOESM9_ESM.pdf]

Figure S6

**a**Zeisel *et al.*, 2015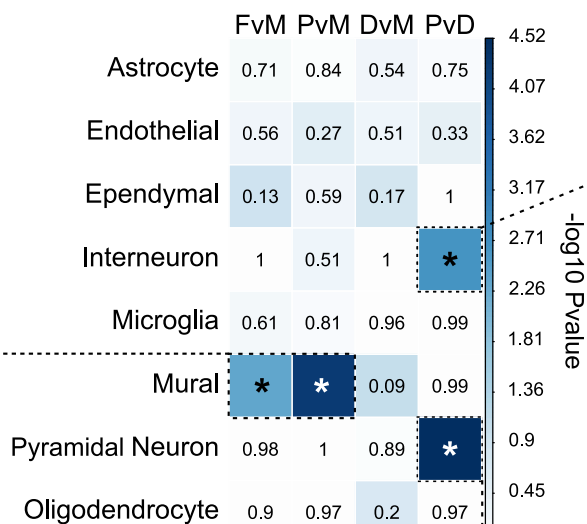**d**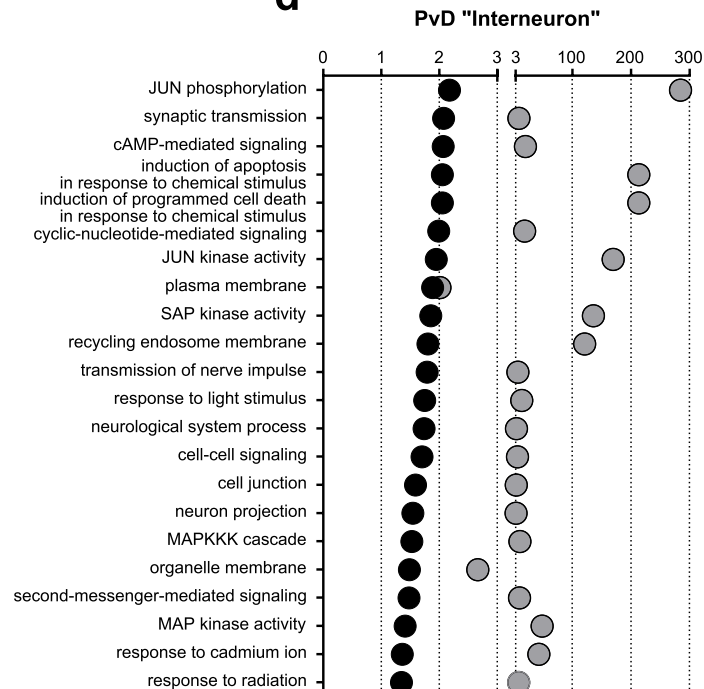**b**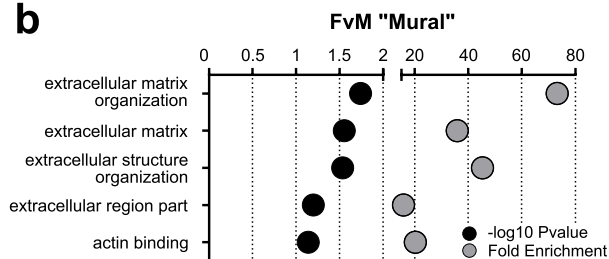**c**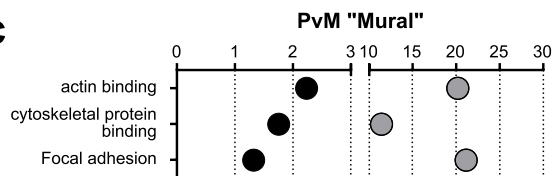**e**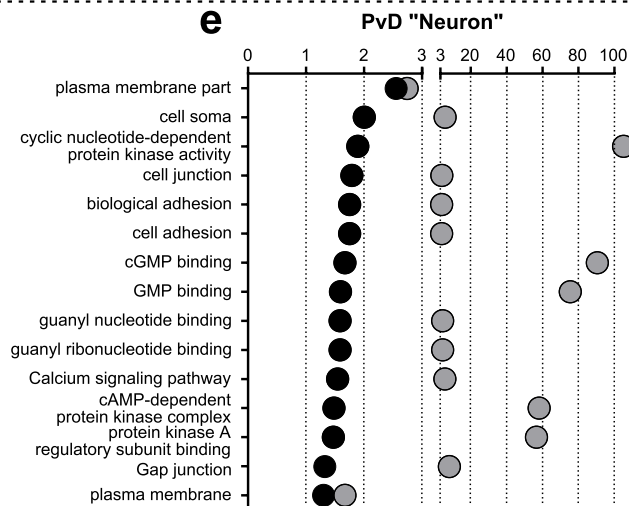

Supplement: Additional file 11: Figure S6. — Cell type enrichment analysis using the Zeisel et al., 2015 dataset. The contribution of each cell type, as defined by the Zeisel et al., 2015 dataset [22], to the profiles of regulations by sex and the estrous cycle was analyzed by testing the enrichment of each term (cell type) in each pairwise comparison. In (a), the matrix depicts the p-value resulting from a one-sided Fisher’s exact test and the background of each cell of the matrix is colored according to the -log10 of this p-value. In (b–e), the corresponding genes for each significantly enriched cell type and pairwise comparison were further analyzed for GO and KEGG database using DAVID. (PDF 354 kb) [file 13059_2015_815_MOESM11_ESM.pdf]

**a**Zhang *et al.*, 2014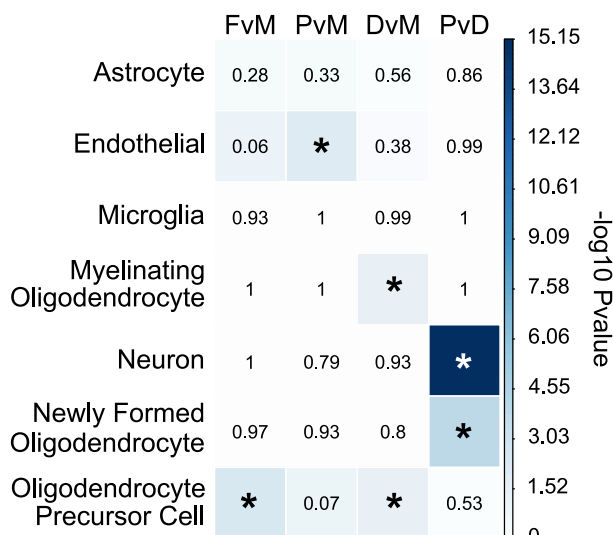**b**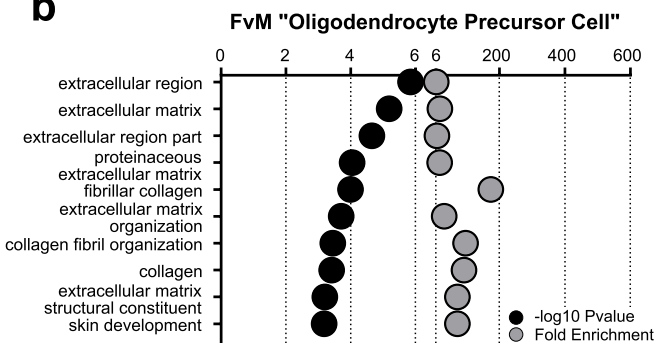**c**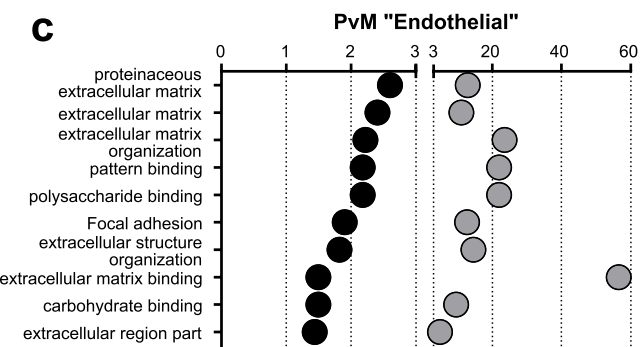**d**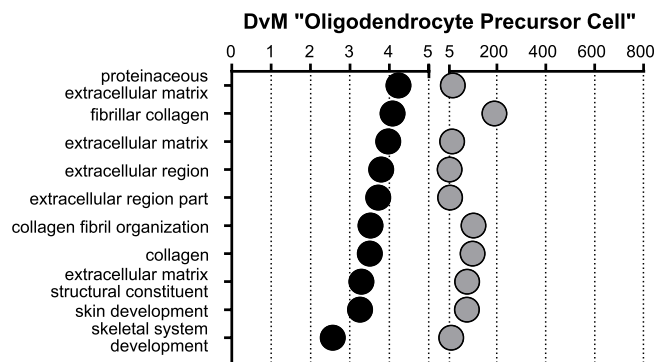**e**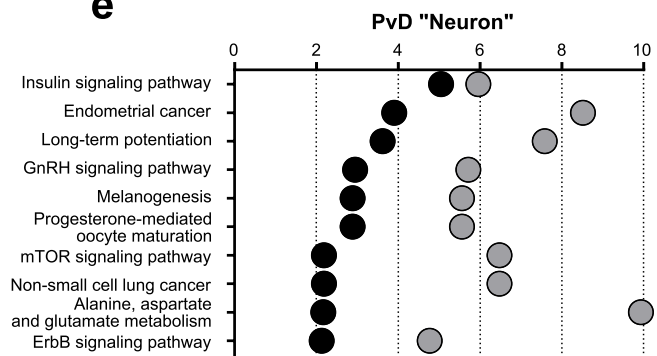**f**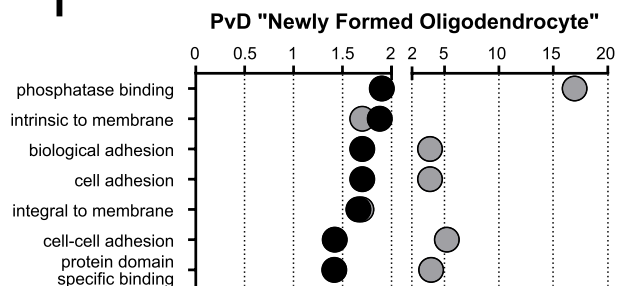

Supplement: Additional file 12: Figure S7. — Cell type enrichment analysis using the Zhang et al., 2014 dataset. The contribution of each cell type, as defined by the Zhang et al., 2014 dataset [42], to the profiles of regulations by sex and the estrous cycle was analyzed by testing the enrichment of each term (cell type) in each pairwise comparison. In (a), the matrix depicts the p-value resulting from a one-sided Fisher’s exact test and the background of each cell of the matrix is colored according to the -log10 of this p-value. In (b–f), the corresponding genes for each significantly enriched cell type and pairwise comparison were further analyzed for GO and KEGG database using DAVID. (PDF 388 kb) [file 13059_2015_815_MOESM12_ESM.pdf]

Figure 6

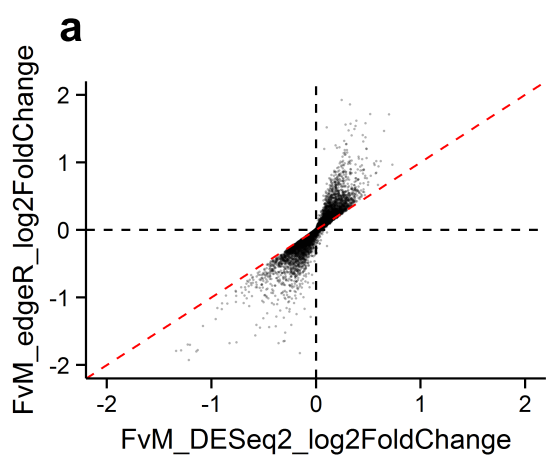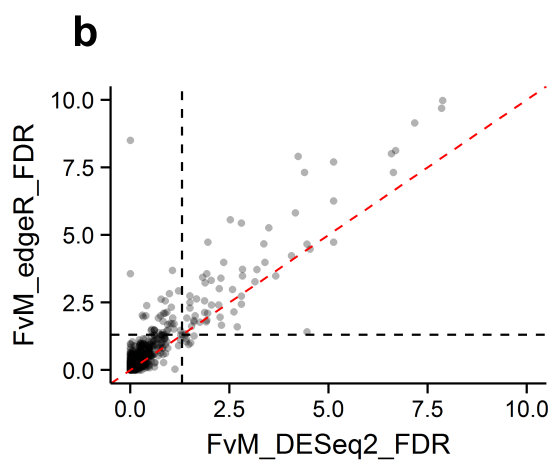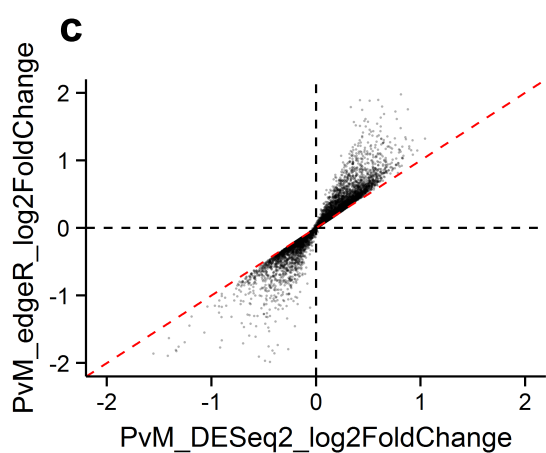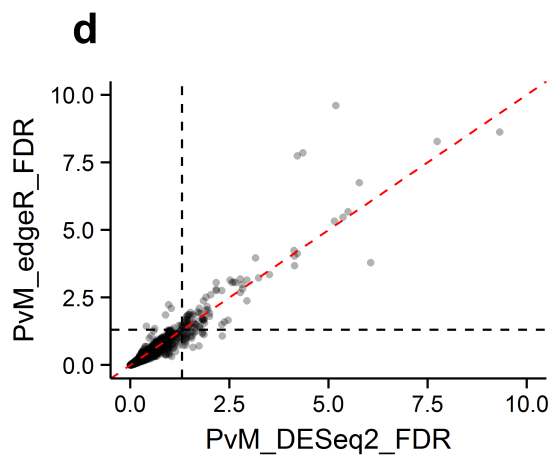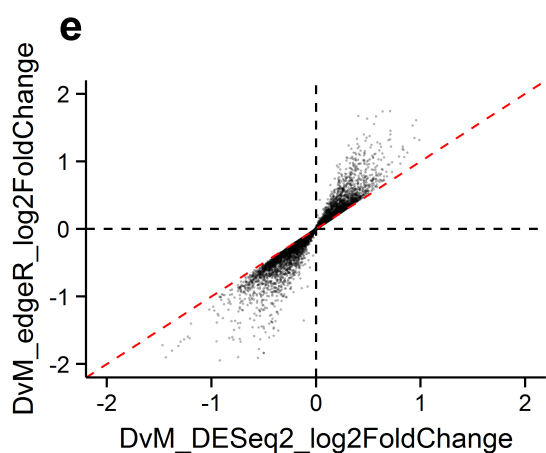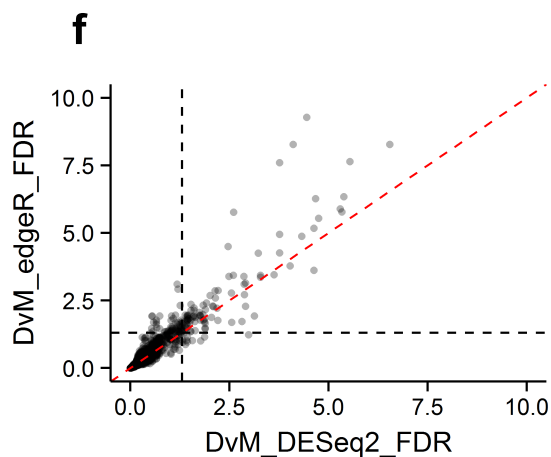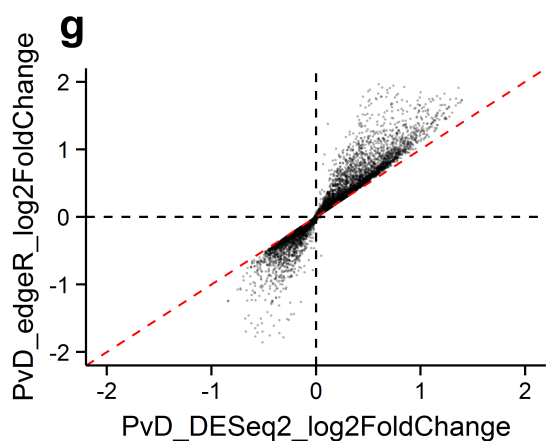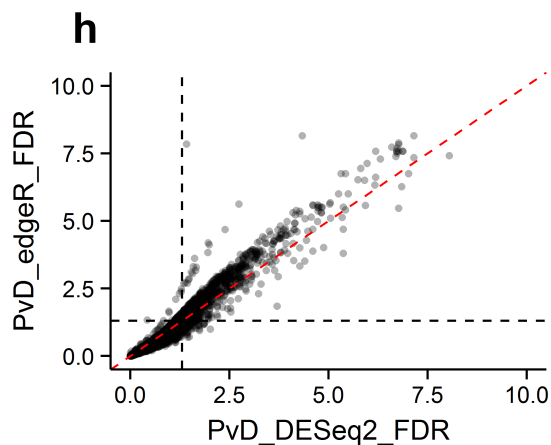

Supplement: Additional file 16: Figure S8. — Comparison of differential expression results from the edgeR and DESeq2 R packages. The log2 of the fold-change (a, c, e, g) and -log10 of the FDR values (b, d, f, h) for each pairwise comparison, females vs males (a, b), proestrus vs males (c, d), diestrus vs males (e, f), and proestrus vs diestrus (g, h) for DESeq2 were plotted against the values obtained from edgeR. In all panels, the red dotted line corresponds to a perfect match between the two packages outputs, while the black dotted lines in (b, d, f, h) correspond to the 5 % FDR threshold. In (a, c, e, g), the majority of fold-changes computed by edgeR were of greater amplitude than those of DESeq2, while in (b, d, f, h), the FDR values computed by DESeq2 tended to exceed those from edgeR, highlighting DESeq2 as more conservative than edgeR on our dataset. (PDF 528 kb) [file 13059_2015_815_MOESM16_ESM.pdf]
